# Flavor variation during the processing of lotus root whole powder: a combination of hot air drying, lysine supplementation and baking

**DOI:** 10.1016/j.fochx.2025.103123

**Published:** 2025-10-06

**Authors:** Chunlan Jia, Yifan Li, Ying Sun, Xueyu Jiang, Hongxun Wang, Kaidi Peng, Yang Yi

**Affiliations:** a College of Food Science and Engineering/Hubei Key Laboratory for Processing and Transformation of Agricultural Products, Wuhan Polytechnic University, Wuhan 430023, China; b Hubei Industrial Technology Research Institute of Jingchu Special Foods, Jingzhou 434000, Hubei, China; c College of Biological and Pharmaceutical Engineering, Wuhan Polytechnic University, Wuhan 430023, Hubei, China

**Keywords:** volatile compounds, differential metabolites, heating processing, amino acid supplementation

## Abstract

To investigate the dynamic changes in flavor characteristics of lotus root whole powder (LRWP) during combined hot-air drying and baking, and the effect of lysine supplementation before baking, volatile compounds and chemical constituents of samples representing different processing stages (raw, hot-air dried, hot-air dried and baked, and hot-air dried with lysine addition and baked) were analyzed using ultraviolet (UV) spectroscopy, electronic nose (*E*-nose), gas chromatography–ion mobility spectrometry (GC-IMS), and ultra-performance liquid chromatography–tandem mass spectrometry (UPLC-MS/MS). Browning intermediates, browning degree, aroma components, and volatile compounds increased progressively with processing, especially during the baking stage. A total of 99 volatile compounds and 586 metabolites were identified, including 49 key volatiles and 345 differential metabolites. Lysine addition promoted the formation of specific volatiles (*e.g.*, 1-octen-3-one, (*Z*)-6-nonen-1-ol, 1-heptanol, linalool oxide) and amino acid-related metabolites, while reducing carbohydrate levels. These results provide insights for enhancing the flavor quality of dried and baked products.

## Highlights

• Flavor changes of lotus root whole powder (LRWP) were studied during hot-air drying and baking with/without lysine.
• 49 key volatile compounds were identified across four processing stages of LRWP.
• 345 differential metabolites were detected during the four processing stages of LRWP.
• Lysine addition before baking enhanced flavor compounds and metabolites in LRWP.

## Graphical abstract

**Figure:**
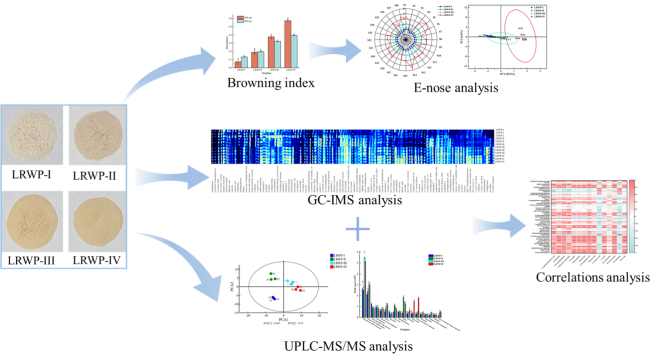

## 1 Introduction

Lotus root (*Nelumbo nucifera* Gaertn.) is a widely consumed aquatic vegetable known for its rich nutritional profile, which includes carbohydrates, phenols, amino acids, vitamins, minerals, and other essential nutrients. It is commonly consumed in both fresh and processed forms, such as starch, slices, and juice. Among products available in the market, lotus root starch is particularly favored by consumers. However, its processing involves steps such as homogenization, filtration, and sedimentation, which can lead to nutrient loss and contribute to environmental pollution due to the generation of wastewater. As a potential alternative, lotus root whole powder (LRWP) produced through peeling, slicing, drying and grinding offers efficient use of resources, low energy consumption in production, and a rich nutrient profile, thereby attracting increasing interest from researchers.

Current researches on LRWP predominantly emphasize its nutritional and antioxidant properties, as well as its applications as a food ingredient. For instance, the study indicated that heated LRWP exhibited higher soluble sugar content, reduced levels of free phenolic compounds, and enhanced antioxidant effects in fruit flies compared to its unheated counterpart (Zhao et al., 2024). Furthermore, it was demonstrated that heat treatment led to a decrease in total starch and resistant starch content, while increasing the amounts of fast digestible and slow digestible starch (Wu et al., 2024). The addition of LRWP into regular-fat model sausages has been shown to enhance shelf-life, reduce lipid oxidation, and suppress microbial growth (Qiu & Chin, 2022). Beyond nutritional and functional attributes, the flavor development of LRWP during thermal processing has received limited attention. Our previous study utilized freeze drying, hot air drying, and hot air drying combined with baking techniques to prepare LRWP, revealing that hot air drying combined with baking produced the most amount of flavor compounds among the three drying methods (Li et al., 2024). However, the mechanisms underlying flavor formation and the evolution of volatile compounds during this process remain unclear.

In addition, volatile compound formation during heat processing is typically associated with lipid oxidation and the Maillard reaction (Song, Du, et al., 2020). The Maillard reaction, which occurs between amino acids and carbohydrates at elevated temperatures, plays a key role in developing unique food flavors, imparting characteristic colors, and enhancing antioxidant activity. Consequently, the addition of exogenous Maillard reaction substrates has been proposed as an effective strategy for improving the flavor quality of baked or dried products. For example, increasing the concentration of amino acids has been shown to darken bread crust, elevate melanoidin content, and enhance the antioxidant activity of white pan bread (Shen et al., 2018). Similarly, the addition of amino acids and sugars has been reported to improve the flavor profile of traditional dried pepper powder (Song, Du, et al., 2020).

Therefore, this study aimed to explore the effects of amino acid supplementation on the sensory characteristics of LRWP during combined hot air drying and baking process. Furthermore, changes in intermediate products, browning intensity, odor characteristics, volatile compounds, and chemical composition were systematically investigated under optimized processing conditions.

## 2 Materials and methods

### 2.1 Reagents

Food-grade glycine (Gly), lysine (Lys), glutamic acid (Glu), alanine (Ala), methionine (Met), valine (Val), arginine (Arg), and isoleucine (Ile) was purchased from Hebei Huayang Biotechnology Co., Ltd. (Wuhan, China). Anhydrous ethanol was obtained from Sinopharm Chemical Reagent Co., Ltd. (Shanghai, China). Methanol and acetonitrile were gained from the Thermo Fisher Scientific. (Shanghai, China).

### 2.2 Samples preparation and collection

Fresh lotus roots (*Cultivar Elian* No. 5) were provided by Wuhan Jinshui-qiliang Agricultural Products Co., Ltd. (Wuhan, China). The roots were cleaned, peeled, and sliced into pieces of uniform thickness (4 ± 1 mm).

#### 2.2.1 Single-factor experimental design

The single-factor experimental treatments were designed as follows: 1) Amino acid type, the lotus root slices were dried in an electric blast drying oven at 70 °C for 3 h, then uniformly sprayed with 20 mL of different amino acid solutions (Lys, Glu, Gly, Arg, Met, Ala, Ile, Val) at a concentration of 0.3 g/L. Subsequently, the slices were baked in an oven with the upper and lower heating temperatures set to 110 °C and 125 °C, respectively, for 10 min; 2) Amino acid concentration: the slices were dried in an electric blast drying oven at 70 °C for 3 h, then uniformly sprayed with 20 mL of a selected amino acid solution at varying concentrations (0.1 g/L, 0.15 g/L, 0.20 g/L, 0.25 g/L, and 0.30 g/L). They were then baked under the same conditions as described above; 3) Timing of amino acid addition: the slices were sprayed with the amino acid solution, prepared at a selected concentration, at three different stages: before hot air drying, during hot air drying, and after hot air drying. All other processing parameters remained consistent with the aforementioned conditions. The untreated slices, without the addition of amino acid, were served as a blank. The dried lotus root slices were ground, passed through an 80-mesh sieve, and stored in a desiccator at room temperature for sensory analysis.

#### 2.2.2 Sensory evaluation

Ten expert assessors, trained in professional sensory evaluation techniques and skilled in describing sensory differences, were selected for the study. The sensory properties of the samples, including appearance, flavor, taste, and texture, were evaluated based on the criteria outlined in Table S1. Appearance was evaluated in both the dried and brewed forms of LRWP, while the other attributes were assessed in the brewed form. To prepare the brewed LRWP, the powder was first dissolved in a small amount of cold water, followed by the addition of boiling water with thorough stirring.

#### 2.2.3 Sample preparation for flavor variation analysis

**Fig. 1:**
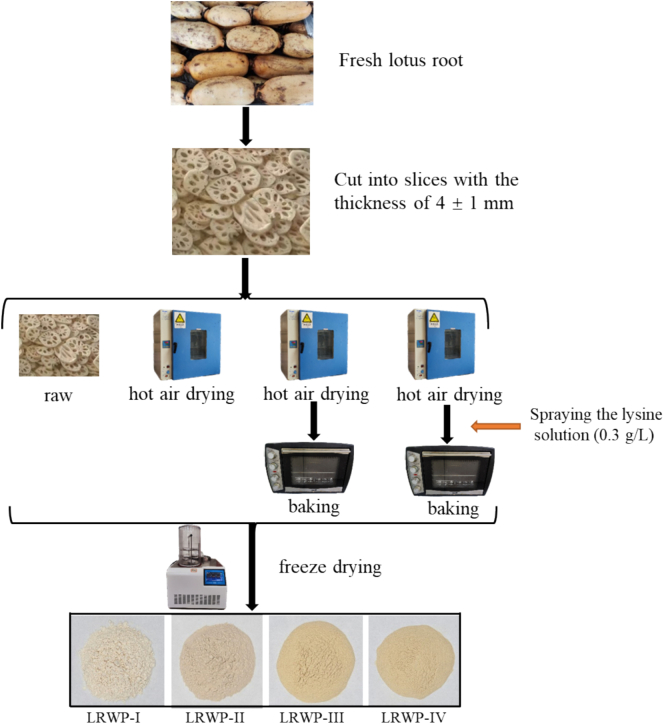
Flow chart of the experiment. Raw, LRWP-I; hot air drying, LRWP-II; hot air drying with baking, LRWP-III; hot air drying with Lys addition followed by baking, LRWP-IV.

Fresh lotus roots were cleaned, peeled, and then sliced into pieces with a uniform thickness of 4 ± 1 mm. Samples were collected at various stages of the drying process (Fig. 1): (1) Raw: fresh lotus root slices; (2) hot air drying stage: fresh lotus root slices were dried in an electric blast drying oven (Shanghai Yiying Network Technology Co., Ltd., Shanghai, China) at 70 °C for 3 h; (3) baking stage without Lys addition: the slices were dried in the same oven at 70 °C for 3 h, followed by baking in an oven (Foshan Beiao Electric Co., Ltd., Guangdong, China) with the upper and lower heating temperatures set to 110 °C and 125 °C, respectively, for 10 min; (4) baking stage with Lys addition: fresh lotus root slices were dried at 70 °C for 3 h, then evenly sprayed with 20 mL of a Lys solution (0.3 g/L), and baked under the same conditions as the stage (3). All four samples collected from stages 1 to 4 were freeze-dried (Ningbo Scientz Biotechnology Co., Ltd., Zhejiang, China) for 48 h to standardize moisture content and were named as LRWP-I, LRWP-II, LRWP-III, and LRWP-IV, respectively. The dried lotus root slices were ground, passed through an 80-mesh sieve, and stored in a desiccator at room temperature for further analysis.

### 2.3 Determination of intermediate products and intensity of browning

The dried sample (1.00 ± 0.01 g) was dissolved in 20 mL of deionized water. This solution was mixed with 3 mL of anhydrous ethanol (95 %, *w*/*w*), followed by centrifugation at 10000 r/min for 10 min. The supernatant was collected for the determination of intermediate product content and browning intensity, measured at wavelengths of 294 and 420 nm, respectively (Y. Hu et al., 2024).

### 2.4 Electronic nose (*E*-nose) analysis

According to the previously reported method (X. Han et al., 2017), *E*-nose analysis was performed using cNose-18 (Shanghai Bosin Industrial Development Co., Ltd., Shanghai, China). Precisely 3.0 ± 0.1 g of LRWP was weighed and incubated in a 50 mL headspace vial. Each measurement was performed in triplicate. The measurement settings were as follows: a pre-heating time of 30 min at 60 °C, a sensor cleaning time of 200 s, a sample measurement time of 200 s, a 1 s interval between measurements, an internal flow rate of 300 mL/min, and a sample injection flow rate of 300 mL/min. Data for analysis were collected between 51 and 55 s. After each measurement, the sensor was thoroughly cleaned, and the instrument baseline was reset.

### 2.5 Gas chromatography-ion mobility spectrometry (GC-IMS) analysis

The volatile flavor compounds were analyzed by GC-IMS [FlavorSpec®, Gesellschaft für analytische Sensorsysteme mbH (G.A.S.), Dortmund, Germany]. 2.0 g of LRWP was transferred to a 20 mL headspace bottle and incubated at 90 °C with shaking for 10 min. Subsequently, 500 μL headspace gas was automatically injected into an FS-SE-54-CB-1 capillary column (15 m × 0.53 mm × 1.0 μm) using an injection needle at 85 °C. High-purity nitrogen (N_2_, 99.999 %) was used as the carrier and drift gas. The flow rate of carrier gas was programmed as follows: an initial flow rate of 2 mL/min for 2 min, then a linear increase to 10 mL/min within 8 min, followed by an increase to 100 mL/min over a period of 10 min, and finally an increase to 150 mL/min over 5 min, which was maintained for 2 min. The flow rate of the drift gas was held constant at 150 mL/min. The temperatures of the drift tube and column were set at 45 °C and 60 °C, respectively. The n-ketones of C_4_-C_9_ were used as external references.

### 2.6 UPLC-MS/MS analysis

The compounds in samples (50 μg) were extracted by homogenizing with 800 μL of pre-cooled methanol (70 %) and 200 μL of internal standard (d3-Leu) in a 1.5 mL Eppendorf tube using a tissue homogenizer (Shanghai Jingxin Industrial Development Co., LTD, Shanghai, China) at 50 Hz for 10 min, followed by ultrasonic treatment in an ice bath at 4 °C for 30 min. Then, the mixture was held at −20 °C for 1 h and subsequently centrifuged at 14000 r/min at 4 °C for 10 min. The supernatant was collected and filtered through a 0.22 μm nylon membrane. 20 μL of the filtrate from each sample was mixed as a quality control (QC) sample to evaluate the reproducibility and stability of the analysis. The filtrate of each sample and the mixed QC sample were transferred into separate 1.5 mL sample bottles for UPLC-MS/MS analysis.

Nontarget metabolomics was conducted using the Waters 2777C Ultra High-Performance Liquid Chromatography system (Waters, Milford, USA) coupled with a Q Exactive HF high-resolution mass spectrometer (Thermo Fisher Scientific, Waltham, USA). The Hypersil GOLD aQ Dim column (2.1 mm × 100 mm, 1.9 μm, Thermo Fisher Scientific, Waltham, USA) was set at 40 °C, and the two mobile phases were composed of: (A) 0.1 % formic acid (*V*/*V*) in distilled water; and (B) 0.1 % formic acid (*V*/*V*) in acetonitrile. The gradient program for the mobile phases was as follows: 0–2 min, 5 % B; 2–22 min, 5 %–95 % B; 22–27 min, 95 % B; 27–27.1 min, 95 %–5 % B; 27.1–30 min, 5 % B. The total flow rate was programmed at 0.3 mL/min and the injection volume of the sample was 5 μL.

The mass spectrometry was carried out using an electrospray ionization source (ESI) in both positive and negative ionization modes. The scanning ranges were 125–1500 *m*/*z* in positive mode and 100–1500 *m*/*z* in negative mode. The primary mass spectrometry resolution was 120,000; AGC (Automatic Gain Control) target was 1e6 for positive mode, and 3e6 for negative mode; the primary maximum ion trap (IT) time was 100 ms. For the secondary mass spectrometry analysis, 3 primary parent ions with the highest intensity were used for the secondary mass spectrometry scan; the resolution was set at 30000, with AGC target of 2e5 in positive mode and 1e5 in negative mode; the IT injection time was 50 ms; and the collision energy (stepped) was set at 20, 30, and 40. The ESI parameters were set as follows: sheath gas flow rate was 40 L/min; aux gas flow rate was 12 L/min; spray voltage of positive and negative ion modes was 3.80 kV and 3.20 kV, respectively; the temperature of the ion transfer capillary was 320 °C; the temperature of the aux gas heater was 350 °C.

### 2.7 Data analysis

All the samples were analyzed at least in triplicate, and the results were shown as “average values ± standard deviations”. One-way analysis of variance was performed by SPSS 22.0 software (IBM Corporation, Armonk, NY, USA) with a significant level of *p* < 0.05. Graphical representations were generated using Origin 2022 software (OriginLab Corporation, Northampton, MA). The chromatograms of GC-IMS were performed using Library Search analysis and Laboratory Analytical Viewer software. PCA, OPLS-DA, and VIP analysis were performed using SIMCA-P 14.1 software (Umetrics, Umea, Sweden). The heatmap was analyzed by an online platform named OmicStudio (https://www.omicstudio.cn). Metabolites were analyzed by Compound Discoverer 3.3 software (Thermo Fisher Scientific, Waltham, USA) and combined with BGI Metabolome, mzCloud and ChemSpider database.

## 3 Results and discussion

The sensory characteristics of LRWP were influenced by the type (Lys, Arg, Gly, Glu, Ala, Met, Val, Ile), concentration (0, 0.1 g/L, 0.15 g/L, 0.20 g/L, 0.25 g/L, 0.30 g/L), and timing of amino acid addition (before, during, or after hot-air drying). As illustrated in the Supplementary Material, the highest overall acceptability of LRWP was achieved when 0.3 g/L of Lys was added after the hot-air drying stage and prior to baking. Therefore, the present study aimed to further investigate the changes in intermediate products, browning intensity, odor characteristics, volatile compounds, and chemical composition of LRWP during processing under this optimized condition.

### 3.1 Changes in browning intermediate products and degree during LRWP processing

**Fig. 2:**
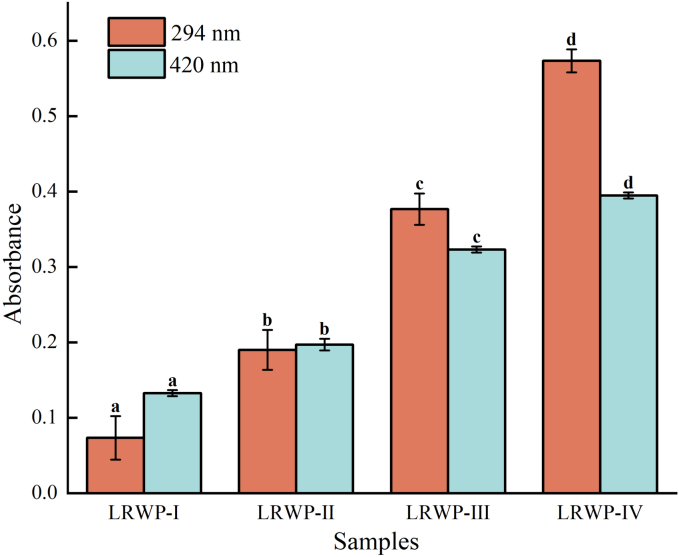
Changes of Maillard intermediates products and browning index in different LRWP samples. Columns of the same color with different letters (a-d) indicate significant differences (*p* < 0.05).

The UV absorbance at 294 nm is commonly used to monitor the formation of intermediate products in the nonenzymatic browning reactions, whereas the browning degree measured at 420 nm serves as an indicator of the late phase of the Maillard reaction (Perusko et al., 2021). In this study, the absorbance values of 294 and 420 nm obtained from four samples were illustrated in Fig. 2, showing a ranking of LRWP-I < LRWP-II < LRWP-III < LRWP-IV. This ranking suggests the progressive formation of intermediate products and the accumulation of browning products during both hot air drying and baking. The observed trends further imply the occurrence of the Maillard reaction and the generation of new substances during the drying of lotus root samples. Additionally, the absorbance values of 294 and 420 nm for LRWP-IV were significantly higher than those for LRWP-III, indicating that the addition of Lys may enhance the Maillard reaction. Similarly, a previous study reported that the Lys content in plums decreased after drying, which may have been due to its reaction with other compounds *via* the Maillard reaction (Michalska et al., 2015).

### 3.2 Changes in *E*-nose characteristics during LRWP processing

**Fig. 3:**
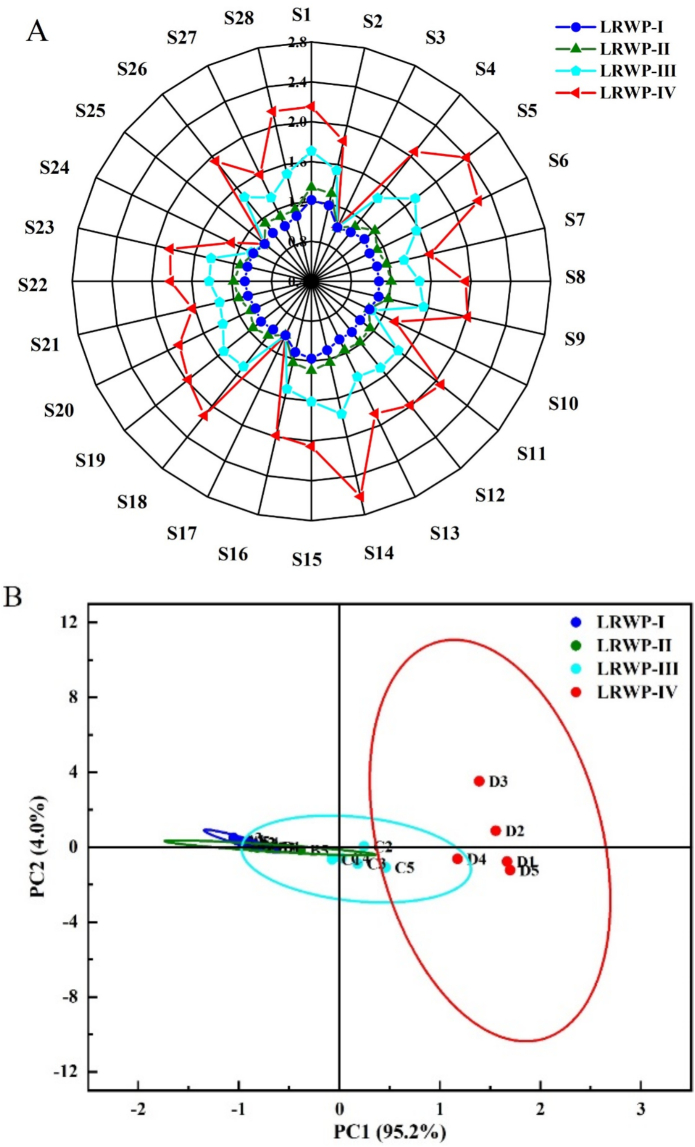
The radar chart (A) and PCA analysis (B) of LRWPs based on the *E*-nose.

As shown in Fig. 3A, the response values of the LRWP-II sample were slightly higher than those of LRWP-I, with both close to 1.2, indicating minimal sensor response (Song, Chen, et al., 2020). Except for the response values from sensors S3 (hydrogen), S10 (hydrogen, hydrogen-containing substance), S17 (hydrogen-containing substance), and S25 (sensitive to alkanes, carbon monoxide, aldehydes, alcohols, nitrogen oxides, ketones, aldehydes, etc), all other values (short-chain alkanes, sulfides, nitrides, carbides, hydrocarbons, some organic solvents, aromatic compounds, volatile organic compounds, aromatic hydrocarbons) for LRWP-IV were higher than those for LRWP-III, as well as for LRWP-I and LRWP-II. This suggested that the increase in odor components in the LRWP predominantly occurred during the baking stage rather than the hot-air drying process, and that the addition of Lys enhanced the formation of volatile components.

Principal component analysis (PCA) of the *E*-nose data was conducted to further elucidate the compositional variables in samples obtained at different drying stages. As shown in Fig. 3B, the first two principal components (PC1: 95.2 % and PC2: 4.0 %) cumulatively accounted for 99.2 % of the total variance, indicating that these two principal components are sufficient to distinguish the differences in the aroma profiles of the four samples (Ke et al., 2022; Song, Chen, et al., 2020). The analysis revealed that LRWP-I and LRWP-II were closely grouped, indicating similar aroma compositions between them. LRWP-I, LRWP-II and LRWP-IV overlapped with LRWP-III, suggesting shared aroma components. In contrast, LRWP-I and LRWP-II were distant from LRWP-IV, suggesting significantly different volatile compound profiles.

### 3.3 Changes in volatile compounds during LRWP processing

#### 3.3.1 GC-IMS topographic plots and volatile fingerprints

**Fig. 4:**
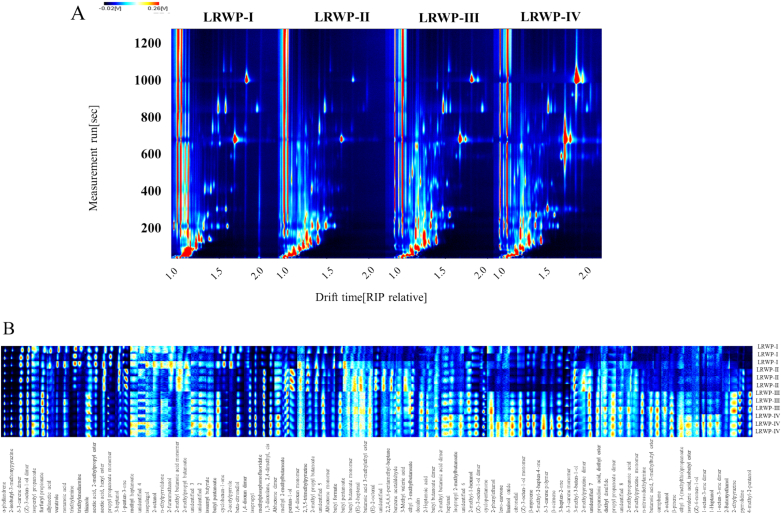
The two-dimensional spectra (A) and fingerprints (B) of volatile compounds from LRWPs.

To investigate the changes in volatile compounds in LRWPs with and without addition of Lys during drying, GC-IMS was performed on four LRWP samples to identify their volatile substances. As shown in GC-IMS topographic plot (Fig. 4A), the vertical axis represents the retention time of the gas chromatograph, and the horizontal axis denotes the ion migration time normalized relative to the reaction ion peak (RIP) position. It was observed that the volatile compounds were effectively separated using GC-IMS technology, showing distinct differences in signal characteristics and intensity among the four LRWP samples. To obtain more intuitional information on these differences, the volatile fingerprints for the four LRWP samples are presented in Fig. 4B. Darker spots indicate lower signal intensity and content, whereas red spots represent compounds with higher concentration.

According to the GC-IMS data mentioned above and its built-in database, a total of 99 volatile compounds were detected in the LRWPs, including 16 alcohols, 5 aldehydes, 7 acids, 24 esters, 12 ketones, 8 akenes, 5 pyrazines, 14 miscellaneous compounds, and 8 unidentified compounds (Table S2). The total number of volatile compounds varied among the four samples, with 46 in LRWP-I, 58 in LRWP-II, 86 in LRWP-III, and 93 in LRWP-IV, indicating an increase in volatile compounds during drying. Specifically, the baking step resulted in a greater increase in the number of detected compounds compared to the hot-air drying step. Additionally, the addition of Lys prior to baking promoted the formation of volatile compounds. These results are consistent with the findings from the *E*-nose analysis.

#### 3.3.2 Flavor compounds

The volatile compounds of LRWP-I included 5 alcohols, 3 aldehydes, 4 acids, 15 esters, 6 ketones, 2 alkenes, 2 pyrazines, and 7 miscellaneous compounds. Most of the substances were also detected in the other three samples, except for several compounds, like butyl formate, cyclohexen-2-one, veratrole, 2-acetylpyrrole, and triethylenediamine. For LRWP-II, the main changes associated with hot air drying were the increase in the contents of 3 alcohols (pentan-1-ol, 3-methyl-3-buten-1-ol, 2-ethyl-1-hexanol), 1 aldehydes (benzene acetaldehyde), 2 acids (3-methyl valeric acid, 2-methylpropanoic acid), 4 esters (2-methylpropyl butanoate, butyl butanoate, propanedioic acid-diethyl ester, propyl propanoate), 1 ketones (cyclopentanone), 2 pyrazines (2-methylpyrazine monomer and dimer), and 4 miscellaneous compounds (1,4-dioxan dimer, 2,2,4,6,6-pentamethyl-heptane, decalin, diethyl disulfide), as compared to LRWP-I.

As far as baking process concerned, LRWP-III had higher contents of 5 alcohols (4-methyl-2-pentanol, 2-phenylethanol, (*Z*)-3-octen-1-ol monomer and dimer, (*Z*)-2-octanol), 1 aldehydes (citronellal), 3 acids (nonanoic acid, allylacetic acid, 2-heptenoic acid), 6 esters (propyl propanoate, isoamyl butyrate, butanoic acid, 3-methylbutyl ester, butyl pentanoate, ethyl 3-(metzhylthio) propanoate, butyl pentanoate), 2 ketones (5-methyl-2-hepten-4-one, 3-hepten-2-one), 6 alkenes (β-myrcene, δ-3-carene, β-ocimene, camphene, δ-3-carene, β-Pinene) and 1 pyrazines (2-ethylpyrazine) in contrast to LRWP-II. Regarding the effect of Lys addition before baking, 3 alcohols ((*Z*)-6-nonen-1-ol, 1-heptanol, linalool oxide), 1 ester (isobutyl isovalerate), and 3 ketones (1-octen-3-one dimer, levo-carvone, 1-octen-3-one monomer) were higher in LRWP-IV than those in the other samples.

#### 3.3.3 Principal component analysis (PCA) of volatile compounds

**Fig. 5:**
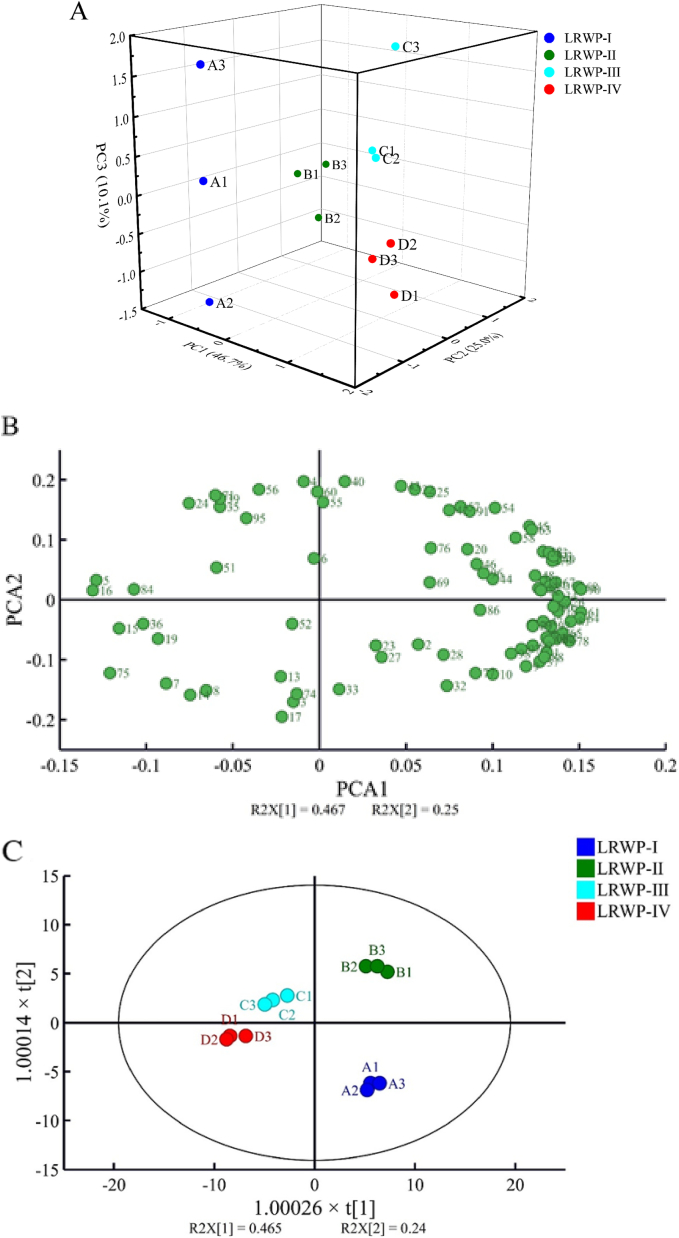

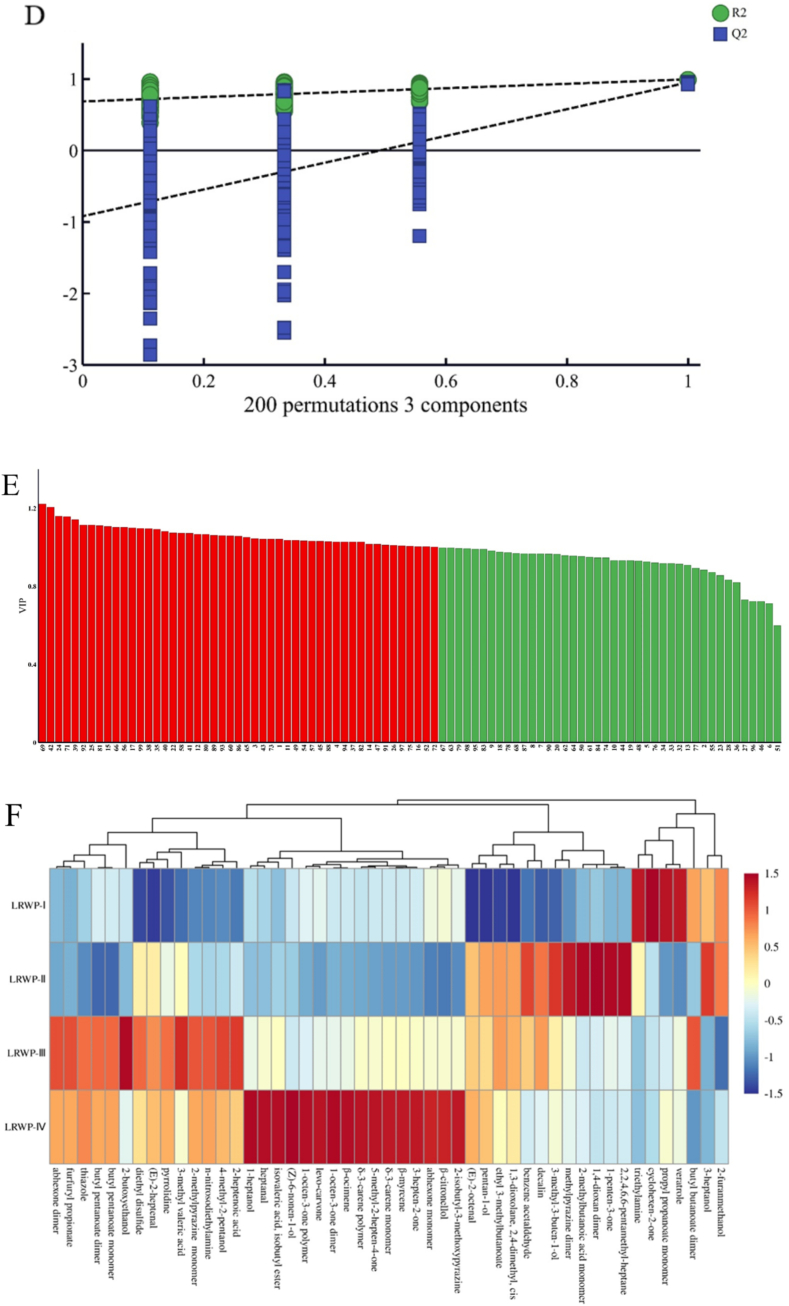
The PCA (A), loading plot (B), OPLS-DA score map (C), the plot of permutations test (D), distribution of VIP values (E), and cluster heatmap (F) of LRWPs based on the volatile compounds analyzed using a GC-IMS method.

Based on the signal peak intensity of volatile compounds obtained by GC-IMS, PCA analysis was performed to further investigate the differences in the volatile compounds of LRWPs from various drying stages. As shown in Fig. 5A, PCA revealed distinct differences in the volatile composition among the samples. The first three principal components, PC1, PC2, and PC3, accounted for 46.7 %, 25.0 %, and 10.1 % of the total variance, respectively, indicating that the data were stable and reliable. The samples were clearly separated and distributed in distinct positions without overlap, highlighting differences in the volatile compounds of LRWPs. The loading plot of volatile compounds was conducted to determine variations among samples (Fig. 5B), effectively showing the correlations between volatile substances and samples (Zhai et al., 2024). It was noticed that LRWP-I exhibited a positive association with volatile compounds such as 2-acetylpyrrole, cyclohexen-2-one, triethylenediamine. LRWP-II was more closely linked to 2-methylbutanoic acid monomer (sweety, cheesy) (Y. L. Qian et al., 2021), 2-methylpyrazine monomer (woody fragrance) (Wang, Duan, & Song, 2024), and 1-penten-3-one (fresh smell) (Mall et al., 2018). For LRWP-III, 2-butoxyethanol and butyl butanoate monomer (fruity aroma) (Djojoputro & Ismadji, 2005) played key roles in its flavor profile. LRWP-IV showed high loadings of (*Z*)-3-octen-1-ol dimer and polymer, citronellal, ethyl 3-(methylthio) propanoate, 1-heptanol, and δ-3-carene polymer, which contributing to fruity, flower, and pine-like aromas (Du et al., 2011; L. Han et al., 2024; Rokonuzzman et al., 2024).

#### 3.3.4 Orthogonal partial least squares-discriminant analysis (OPLS-DA) analysis of volatile compounds

PCA analysis was utilized to elucidate trends in sample classification, while orthogonal partial least squares-discriminant analysis (OPLS-DA) was employed as a supervised multivariate statistical method to identify the variables influencing the differences between groups (Zhai et al., 2024). The OPLS-DA score map (Fig. 5C) differentiates samples across various drying stages, with the two principal components accounting for 70.5 % of the total variable. The relative distances observed in the score plot signify the magnitude of differences in volatile compositions among samples. Specifically, LRWP-I was positioned in the lower-right quadrant, LRWP-II in the upper-right quadrant, and both LRWP-III and LRWP-IV occupied the left quadrant. This distribution indicates distinct volatile profiles among the four LRWPs. Furthermore, despite the close proximity between LRWP-III and LRWP-IV, the distance between LRWP-IV and LRWP-II was greater than that between LRWP-III and LRWP-II, indicating more pronounced relative differences between LRWP-IV and LRWP-II.

Additionally, a 200-permutation test was conducted to validate the practicability of predictability of the established OPLS-DA model. As presented in Fig. 5D, the fit parameters (R^2^X), model explanatory power (R^2^Y), and predictive power (Q^2^) were 0.899, 0.984, and 0.901, respectively. Furthermore, the Q^2^ and R^2^ values on the left side of the graph, representing the values from the permutation test stochastic models, were significantly lower than those of the original model on the right. These findings confirm that the OPLS-DA model is reliable without overfitting.

To evaluate the contribution of each variable in the OPLS-DA model and identify the volatile characteristic markers distinguishing LRWPs at different drying stages, the variable importance in the projection (VIP) values were presented in Fig. 5E. Compounds with VIP ≥ 1, highlighted in red, are generally regarded as potential crucial volatile substances, with higher VIP values indicating more significant differences between LRWP samples. The total number of these compounds was 49, including 8 alcohols, 4 aldehydes, 3 acids, 7 esters, 9 ketones, 4 alkenes, 5 pyrazines, and 9 miscellaneous. Alcohols may be formed through lipid oxidation and Strecker degradation reactions, contributing to characteristic flavor profiles during thermal processing(M. Qian et al., 2020). Aldehydes are primarily generated from lipid oxidation and the Strecker degradation of amino acids (Lee et al., 2021). Variations in acid content during heat processing may be attributed to lipid degradation, oxidation, Maillard reactions, or volatilization of low molecular weight acids. Esters are typically formed through condensation reactions between alcohols and acids, especially under heat-induced conditions. Ketones are commonly derived from Maillard reactions or formed as a result of lipid degradation, oxidation, and subsequent thermal reactions (Domínguez et al., 2019). Alkenes are susceptible to oxidation reactions during thermal treatment, which can lead to changes in flavor characteristics (Okonkwo et al., 2024). Pyrazines, which impart roasted and earthy aromas, are mainly produced through Maillard reactions and cellular structure disruption during high-temperature processing.

To further analyze the differences, a heat map analysis was performed based on the peak intensity values of each volatile compound marker (Fig. 5F). LRWP-I was characterized by key discriminant compounds such as veratrole, propyl propanoate monomer, cyclohexen-2-one, and triethylamine. In contrast, LRWP-II exhibited higher levels of compounds like 2,2,4,6,6-pentamethyl-heptane, 1-penten-3-one, 1,4-dioxan dimer, 2-methylbutanoic acid monomer, methylpyrazine dimer, 3-methyl-3-buten-1-ol, and benzene acetaldehyde compared to other samples. LRWP-III contained relatively higher concentrations of 2-heptenoic acid, 4-methyl-2-pentanol, n-nitrosodiethylamine, ethylpyrazine, 3-methyl valeric acid, pyrrolidine, (*E*)-hept-2-enal, diethyl disulfide, 2-butoxyethanol, butyl pentanoate monomer, butyl pentanoate dimer, thiazole, furfuryl propionate, and abhexone dimer. LRWP-IV was distinguished by the highest concentrations of several compounds, including 2-isobutyl-3-methoxypyrazine, β-citronellol, abhexone monomer, 3-hepten-2-one, β-myrcene, δ-3-carene polymer, 5-methyl-2-hepten-4-one, β-ocimene, 1-octen-3-one dimer, levo-carvone, 1-octen-3-one polymer, (*Z*)-6-nonen-1-ol, isovaleric acid isobutyl ester, 3-heptanal, and 1-heptanol, compared to the other samples.

### 3.4 Changes in chemical metabolites during LRWP processing

#### 3.4.1 Comparison on the chemical metabolites of LRWPs

**Fig. 6:**
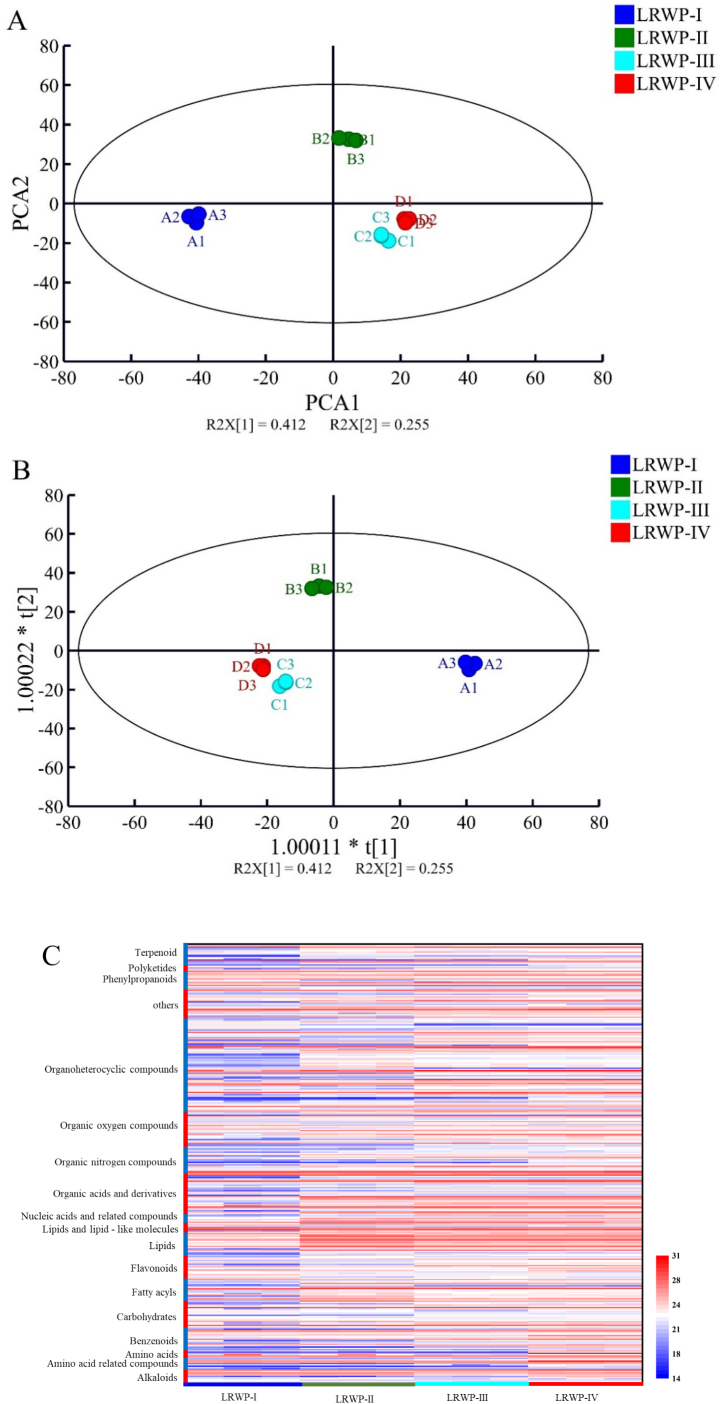

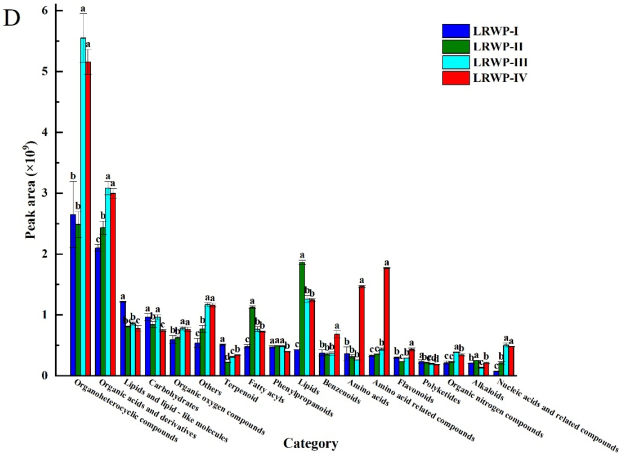
The PCA plot (A), OPLS-DA score map (B), hierarchical cluster analysis (C), and comparison on the differential metabolites of LRWPs based on the non-targeted metabolomics.

To investigate the chemical changes of LRWPs occurring at various drying stages, a non-targeted metabolomics approach using UPLC-MS/MS was employed to characterize the metabolite profiles of four different samples. The analysis collected 495 and 1095 mass spectrometric features from the negative and positive ion modes, respectively, including 177 and 409 metabolites that were identified through qualitative matching analysis. PCA revealed a clear separation of all samples within a 95 % confidence interval (Fig. 6A), with the PC1 of 41.2 % and the PC2 of 25.5 %, highlighting notable differences in chemical composition among the four samples. Furthermore, OPLS-DA (Fig. 6B) confirmed a distinct separation among the four samples, with cross-validation R^2^Y and Q^2^ values of 0.995 and 0.967, respectively. This suggests that hot air drying, baking, and the addition of Lys before baking significantly influenced the compositional changes in LRWP during drying.

345 differential feature metabolites were screened with VIP > 1, *p* < 0.05, and FC > 1.50 or FC < 0.66, including 75 organoheterocyclic compounds, 36 organic acids and derivatives, 27 organic oxygen compounds, 21 carbohydrates, 21 organic nitrogen compounds, 20 lipids, 19 flavonoids, 18 benzenoids, 17 fatty acyls, 13 terpenoids, 13 phenylpropanoids, 11 lipids and lipid-like molecules, 9 amino acid-related compounds, 9 alkaloids, 7 nucleic acids and related compounds, 6 amino acids, 4 polyketides, and 19 others (Table S3). As the drying process progresses, the number of increased differential metabolites exceeds that of decreased ones. The addition of Lys prior to the baking stage significantly altered the composition of LRWP. Moreover, hierarchical cluster analysis (HCA) (Fig. 6C) revealed distinct differences among the four samples, highlighting the dynamic changes in differential metabolites throughout the drying processes.

**Fig. 7:**
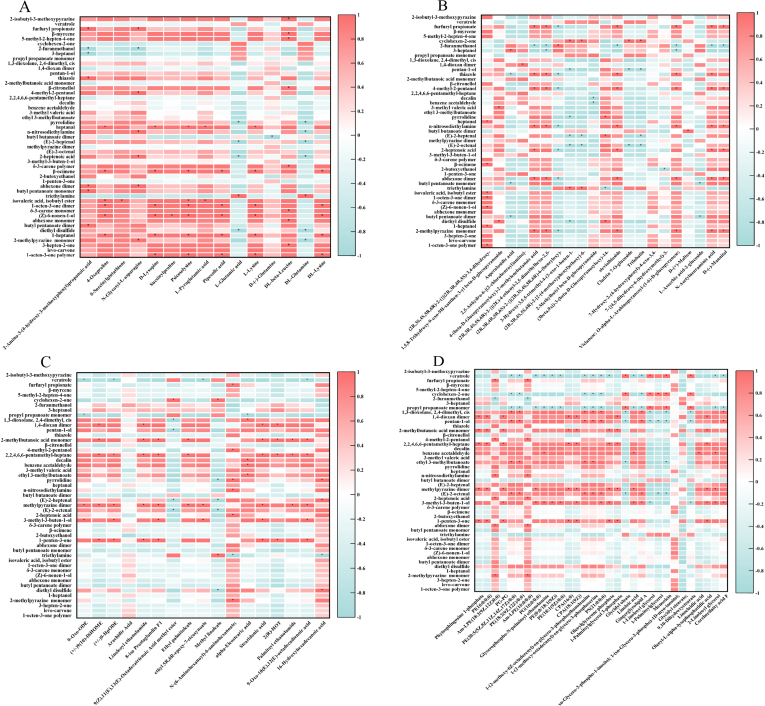
Correlation analysis between partial volatile compounds and amino acid (A), carbohydrates (B), fatty acyls (C), lipids and lipid-like molecules (D).

As shown in Fig. 6D, organoheterocyclic compounds, organic acids and derivatives, and lipids and lipid-like molecules constituted the majority of the differential metabolites identified. These findings are consistent with those of the previous study, which demonstrated that these three super classes constitute the most abundant common differential metabolites in *Cordyceps sinensis* when subjected to various drying methods, including freeze-drying, oven-drying, and air-drying (Xiao et al., 2024). Notably, organoheterocyclic compounds exhibited a relatively stable trend during the hot air drying phase, yet exhibited a substantial increase following the baking process. Organic acids and derivatives demonstrated a consistent upward trend throughout drying. Lipids and lipid-like molecules primarily experienced a decline during the hot air drying stage, followed by a stabilization period. Conversely, fatty acyls and lipids increased during the hot air drying phase before decreasing during baking. Organic oxygen compounds constituted a significant fraction of the identified differential metabolites, with their total relative content remaining unchanged during the hot air drying process but showing an increase during the baking stage.

Various terpene compounds are known to contribute to plants' unique aroma and nutritional properties, offering benefits such as anti-inflammatory, anti-cancer, and antioxidant effects (Lowe et al., 2021). These compounds are commonly utilized in cosmeceuticals and as flavor additives in the food industry. In this study, we observed the relative content of terpenoids decreased during the hot air drying stage and kept slight increase during the baking stage. These changes in terpenoid levels may be attributed to the oxidation of terpenes into terpenoids and the vaporization of terpenoids at elevated temperatures (Chang et al., 2021). The other differential metabolites, such as benzenoids displayed a significant increase in LRWP when Lys was added before the baking. Polyketides exhibited a gradual decreasing trend, while organic nitrogen compounds, as well as nucleic acids and their related compounds, showed a continuous increase during both the hot air drying and baking stages. In contrast, alkaloids increased initially during hot air drying but showed a decline during the baking stage.

#### 3.4.2 Effect of drying processes on carbohydrates, amino acids and phenolic compounds of LRWPs

Lotus root is a rich source of carbohydrates, which not only contribute to its natural sweetness but also present potential for use in food production, particularly in bakery products. The total relative content of carbohydrates among the four samples followed the order: LRWP-IV < LRWP-II < LRWP-I ≤ LRWP-III (Fig. 6D). This trend suggests that carbohydrate levels declined during the hot air drying stage, increased during the subsequent baking stage in the absence of Lys, but continued to decline when Lys was added prior to baking. Some sugars may participate in the Maillard reaction during processing, leading to the development of characteristic aromas in the final product. Among the 21 selected carbohydrates (Table S3), 5 were found to be increased and 6 decreased during hot air drying; similarly, 6 carbohydrates were increased and 8 decreased when comparing LRWP-III to LRWP-II, while 8 were increased and 4 decreased when comparing LRWP-IV to LRWP-II. Additionally, both D-(−)-mannitol and steviolbioside exhibited continuous increases throughout the hot air drying and baking processes. Carbohydrates such as *N*-acetylneuraminic acid and vicianose showed increases during the baking process, while other compounds, such as asperulosidic acid, and L-ascorbic acid-2-glucoside were found to decrease during the baking stage. Previous studies have reported that hot air drying can significantly reduce the sugar content in foods like sweet corn and mushrooms, and this reduction may be attributed to the Maillard reaction or sugar degradation (S. Hu et al., 2020; Yao et al., 2021). Conversely, the increase in some sugars may be the result of the breakdown of cell wall components, such as cellulose and hemicellulose (Zhou et al., 2021).

Amino acids also contribute to the formation of unique flavors in food and can react with sugars through the Maillard reaction, leading to the formation of new compounds (Michalska et al., 2015). In this study, the supplementation of Lys resulted in a significant increase in the content of amino acids and amino acid-related compounds in LRWP-IV, compared to LRWP-III (Fig. 6D). Furthermore, 6 amino acids were identified among the differential metabolites across four samples (Table S3). Specifically, the content of L-glutamic acid decreased during both hot air drying and baking stages. D-(−)-glutamine content increased during the hot air drying stage but decreased during the baking stage in the absence of Lys addition.

Phenolic compounds in lotus root are considered key contributors to its antioxidant activity. Among the differential metabolites, flavonoids and phenylpropanoids were the dominant phenolic compounds (Table S3). During the hot air drying process, 15 phenolic substances showed a significant decrease, while only 4 phenolic compounds exhibited an increase. However, during the baking process, more phenolic substances were found to increase rather than decrease. Specifically, compared to LRWP-II, 9 and 12 phenolic compounds were increased in LRWP-III and LRWP-IV, respectively, while 7 phenolic compounds were decreased in both cases. This phenomenon could be attributed to the high temperature and long exposure during hot air drying, which lead to the oxidation and degradation of polyphenols, resulting in a reduction of phenolic content (Qu et al., 2018). Conversely, flavonoids such as naringenin, quercetin, sideretin, and isorhamnetin showed increased levels after the baking. This increase could be due to the inactivation of polyphenol oxidase during hot air drying and the release of bound phenolic compounds (Hayat et al., 2010; Prathapan et al., 2009).

#### 3.4.3 Effect of Lys addition on the chemical metabolites of LRWPs

When comparing LRWP-IV to LRWP-III, a total of 80 differential metabolites were identified (VIP > 1, *p* < 0.05, and FC > 1.50 or FC < 0.66), with 60 metabolites being increased and 20 decreased. Notably, several increased compounds, including *N*-methyltryptamine, pipecolic acid, spermidine, benzoic acid, salicylic acid, 1-pyrenol, and pinocembrin, are known to contribute to the development of distinctive flavors in food. Specifically, *N*-methyltryptamine, D-lysopine, pipecolic acid, L-Lys, spermidine, and skatole were linked to amino acid metabolism and exhibited higher levels in LRWP-IV compared to LRWP-III, while oxalosuccinate, alpha-d-glucose-1,6-bisphosphate, and (2*R*)-2,3-dihydroxypropanoic acid, involved in carbohydrate metabolism, were decreased in LRWP-IV relative to LRWP-III.

### 3.5 Correlations between key volatile compounds and differential metabolites in LRWPs

Amino acids, carbohydrates, fatty acyls, and lipid-related compounds are known to participate in various chemical transformations during food processing, including enzymatic reactions, thermal degradation, Maillard reactions, and oxidative processes induced by heating (Zhao et al., 2023). These transformations contribute significantly to the formation of volatile flavor compounds (L. Zhang et al., 2021). In the present study, correlation analysis was performed to investigate the potential associations between key volatile flavor compounds and these precursor substances.

As illustrated in Fig. 7A, diethyl disulfide, 2-heptenoic acid, (*E*)-2-heptenal, and pyrrolidine were found to exhibit significant negative correlations with L-glutamic acid and DL-glutamine (*p* < 0.05), whereas triethylamine displayed a significant positive correlation (*p* < 0.05). In addition, compounds such as 1-octen-3-one polymer/dimer, 1-heptanol, (*Z*)-6-nonen-1-ol, β-ocimene, and heptanal were positively associated with N^2^-(D-1-carboxyethyl)-L-lysine, psicoselysine, and L-lysine (*p* < 0.05). L-Lysine can act as a precursor for the formation of carboxymethyllysine (CML) and carboxyethyllysine (CEL) *via* Maillard reaction pathways, through its reaction with reactive carbonyl intermediates such as glyoxal and methylglyoxal (Quan et al., 2020). Interestingly, the sample group LRWP-IV exhibited elevated levels of N^2^-(D-1-carboxyethyl)-L-lysine alongside reduced levels of D-(+)-maltose compared to LRWP-III, suggesting the possibility that Lys may engage in Maillard reactions with maltose to generate volatile compounds.

Further correlation analysis (Fig. 7B) revealed that several volatile compounds were positively correlated with 4-(β-D-glucopyranosyloxy)-2-methylenebutanoic acid, steviolbioside, *N*-acetylneuraminic acid, and D-(−)-mannitol, whereas negative correlations were observed with cladrin 7-O-glucoside and trilobatin.

As presented in Fig. 7C, volatile compounds including 1-penten-3-one, 3-methyl-3-buten-1-ol, methylpyrazine dimer, 2,2,4,6,6-pentamethyl-heptane, 2-methylbutanoic acid monomer, and 1,4-dioxane dimer showed positive correlations with specific fatty acyls. Notably, methylpyrazine dimer was positively correlated with (+/−)-9(10)-DiHOME, linoleoyl ethanolamide, 8-iso prostaglandin F1, N-(6-aminohexanoyl)-6-aminohexanoate, stearidonic acid, and 9-oxo-10(*E*),12(*E*)-octadecadienoic acid. Conversely, the majority of volatile compounds demonstrated negative correlations with 9(*Z*),11(*E*),13(*E*)-octadecatrienoic acid methyl ester and methyl linoleate.

A similar trend was observed in lipid-related compound analysis (Fig. 7D), where 1-penten-3-one, 3-methyl-3-buten-1-ol, (*E*)-2-octenal, methylpyrazine dimer, 2,2,4,6,6-pentamethyl-heptane, and 2-methylbutanoic acid monomer were positively associated with a range of lipid and lipid-like molecules. In contrast, propyl propanoate monomer and veratrole showed negative correlations. It was reported that linoleic acid could be oxidized by lipoxygenase or dioxygenase and then cleaved with hydroperoxide lyase to produce 3-octen-1-ol and 1-octen-3-one (Y. Zhang et al., 2023). In the present work, a decrease in linoleic acid content was observed in lysine-supplemented samples during the baking stage, accompanied by an increase in 1-octen-3-one levels (*p* > 0.05), suggesting potential involvement of linoleic acid in volatile compound generation. Moreover, linoleic acid degradation may also yield (*E*)-2-heptenal (Wang, Chen, et al., 2024). Although the current study revealed a positive but non-significant correlation between (*E*)-2-octenal and linoelaidic acid (*p* > 0.05), a significant positive association (*p* < 0.01) was previously reported by (Duan et al., 2024).

## 4 Conclusions

In the present study, the flavor characteristics of LRWP at different processing stages during a combination of hot air drying and baking, with and without Lys supplementation before baking, were analyzed using UV detection, *E*-nose, GC-IMS, and UPLC-MS/MS. The results indicated that intermediate products, browning intensity, odor components, and volatile compounds all increased during both the hot air drying and baking processes, particularly when Lys was added. A total of 99 volatile compounds were detected across the four LRWP samples with 49 key volatile substances (VIP ≥ 1) identified through GC-IMS analysis, highlighting significant variations in flavor profiles due to the combined effects of hot air drying, Lys supplementation, and baking.

Furthermore, 586 metabolites were identified by UPLC-MS/MS analysis, among which 345 differential metabolites were screened (VIP > 1, *p* < 0.05, and FC > 1.50 or FC < 0.66). Among the four LRWP samples, LRWP-I exhibited the highest levels of lipids, lipid-like molecules, and terpenoid compounds. LRWP-II was characterized by the highest concentrations of fatty acyls and lipid compounds. In contrast, both LRWP-III and LRWP-IV contained elevated levels of organoheterocyclic compounds, organic acids, and their derivatives. The addition of Lys in LRWP-IV resulted in increased levels of amino acids and amino acid-related metabolites, accompanied by a reduction in carbohydrate content compared to LRWP-III. Correlation analysis revealed both positive and negative associations between volatile compounds and various classes of metabolites, including amino acids, carbohydrates, fatty acyls, and lipid-related substances. This study provides clear evidence that hot air drying, Lys supplementation, and baking significantly alter the volatile compounds and chemical composition of LRWP. These changes are likely associated with Maillard reactions, thermal degradation, and oxidative processes. However, the formation of numerous metabolites and the complexity of the underlying reactions warrant further investigation to elucidate the precise pathways and mechanisms involved. Overall, these findings offer valuable insights into flavor formation mechanisms and suggest a promising processing strategy to enhance quality control and broaden the potential application of LRWP-based products.

## CRediT authorship contribution statement

**Chunlan Jia:** Writing – original draft, Investigation, Conceptualization. **Yifan Li:** Writing – original draft, Investigation, Formal analysis. **Ying Sun:** Methodology, Data curation. **Xueyu Jiang:** Formal analysis, Data curation. **Hongxun Wang:** Project administration, Investigation. **Kaidi Peng:** Writing – original draft, Methodology, Formal analysis, Conceptualization. **Yang Yi:** Writing – review & editing, Validation, Supervision, Funding acquisition.

## Declaration of competing interest

The authors declare that they have no known competing financial interests or personal relationships that could have appeared to influence the work reported in this paper.

## Data Availability

Data will be made available on request.

## Appendix A Supplementary data

Supplementary material Image 1
