# Supplementary material for: Flavor variation during the processing of lotus root whole powder: a combination of hot air drying, lysine supplementation and baking
Source: Food Chem X. 2025 Oct 6;31:103123. doi: 10.1016/j.fochx.2025.103123 (PMC12547767; doi:10.1016/j.fochx.2025.103123)
Supplement: Supplementary file 1 — Supplementary material [file mmc1.docx]

**Supplementary Material**

**1. Sensory analysis**

The total sensory scores of LRWP with various amino acid additions followed this order: Lys > Blank > Arg > Gly > Glu > Ala > Met > Val > Ile (Fig. S1A). Lys addition achieved the highest total score of 87 points, with the highest scores in appearance, flavor, texture, and overall appearance, although its taste was relatively poor. Arg supplementation scored highest in taste, but its scores for other sensory characteristics were lower than those of Lys supplementation or the Blank. Additionally, LRWP supplemented with Ile, Met, or Val exhibited noticeable unpleasant and pungent odors, resulting in lower sensory scores. In conclusion, the addition of Lys provided the best overall sensory quality for LRWP compared to other amino acids or the Blank. Therefore, LRWP supplemented with Lys was selected for further study.

Lys at a concentration of 0.3 g/L achieved the highest scores for flavor, taste, appearance, texture, and total sensory points, which were significantly higher than those of other sample groups (Fig. S2B). Therefore, adding Lys at a concentration of 0.3 g/L results in superior sensory quality. The sensory score radar diagram for different Lys addition timings is shown in Fig. S1C. The total sensory scores were ranked as follows: after hot air drying > during hot air drying > before hot air drying. LRWP achieved the highest total sensory score, as well as the highest scores for taste and flavor, when Lys was added after hot air drying. Additionally, the appearance scores were no significant difference for the three treatments in dried samples, although the appearance scores for the before and after hot air drying groups in the brewed LRWP were higher than those for the during hot air drying stage. Overall, adding Lys after hot air drying contributed more significantly to the sensory quality of LRWP, particularly in terms of flavor.

**2. Supplementary Figures**

**Fig.S1.** Effects of amino acid type (A), concentration (B), addition timing (C) on the sensory scores of LRWPs.

**3. Supplementary Tables**

**Table S1.** Sensory evaluation criteria of LRWP.

**Table S2.** Volatile compounds at different processing stages.

**Table S3.** Differential feature metabolites at different processing stages.

**Fig.S1**


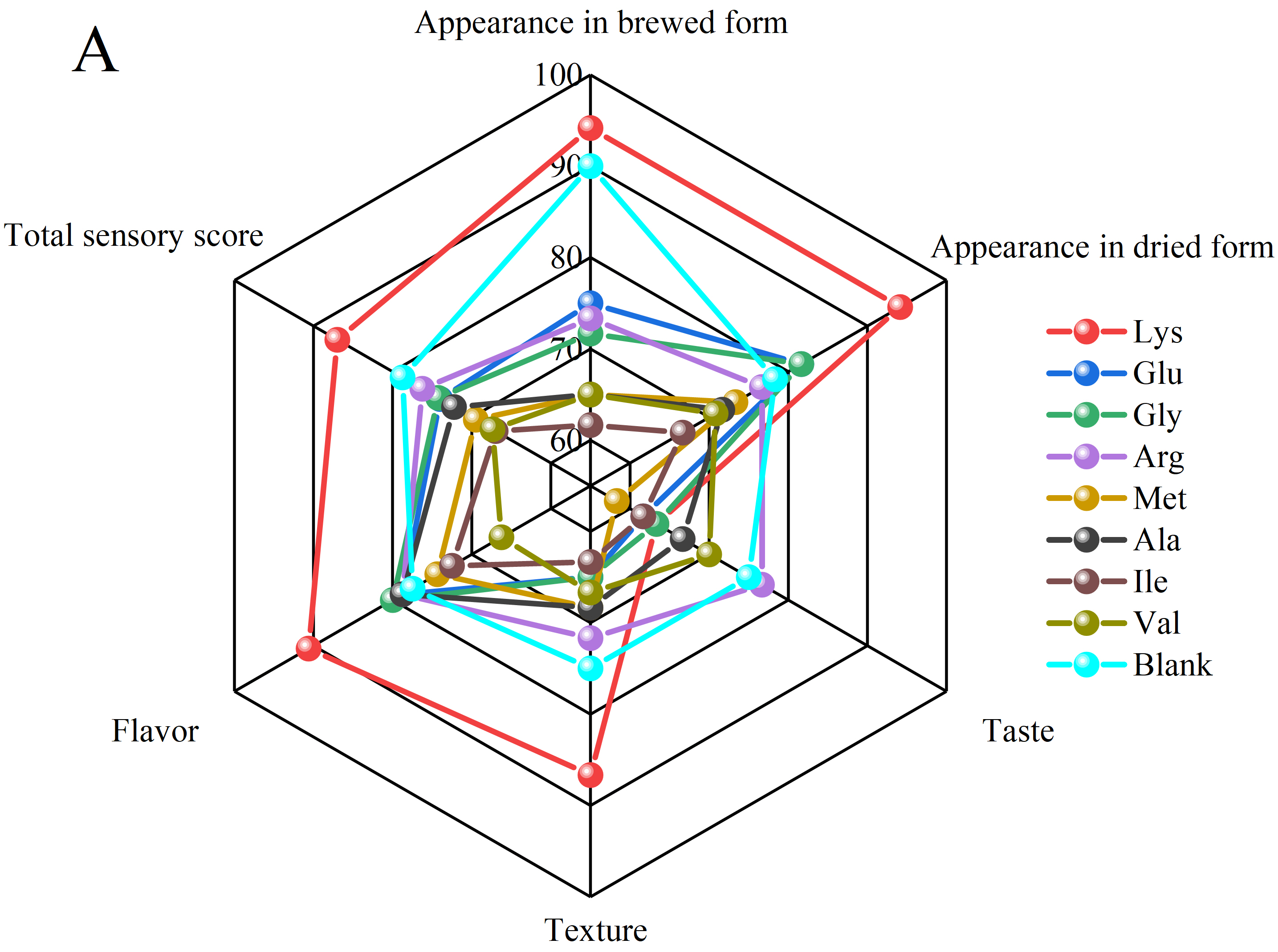

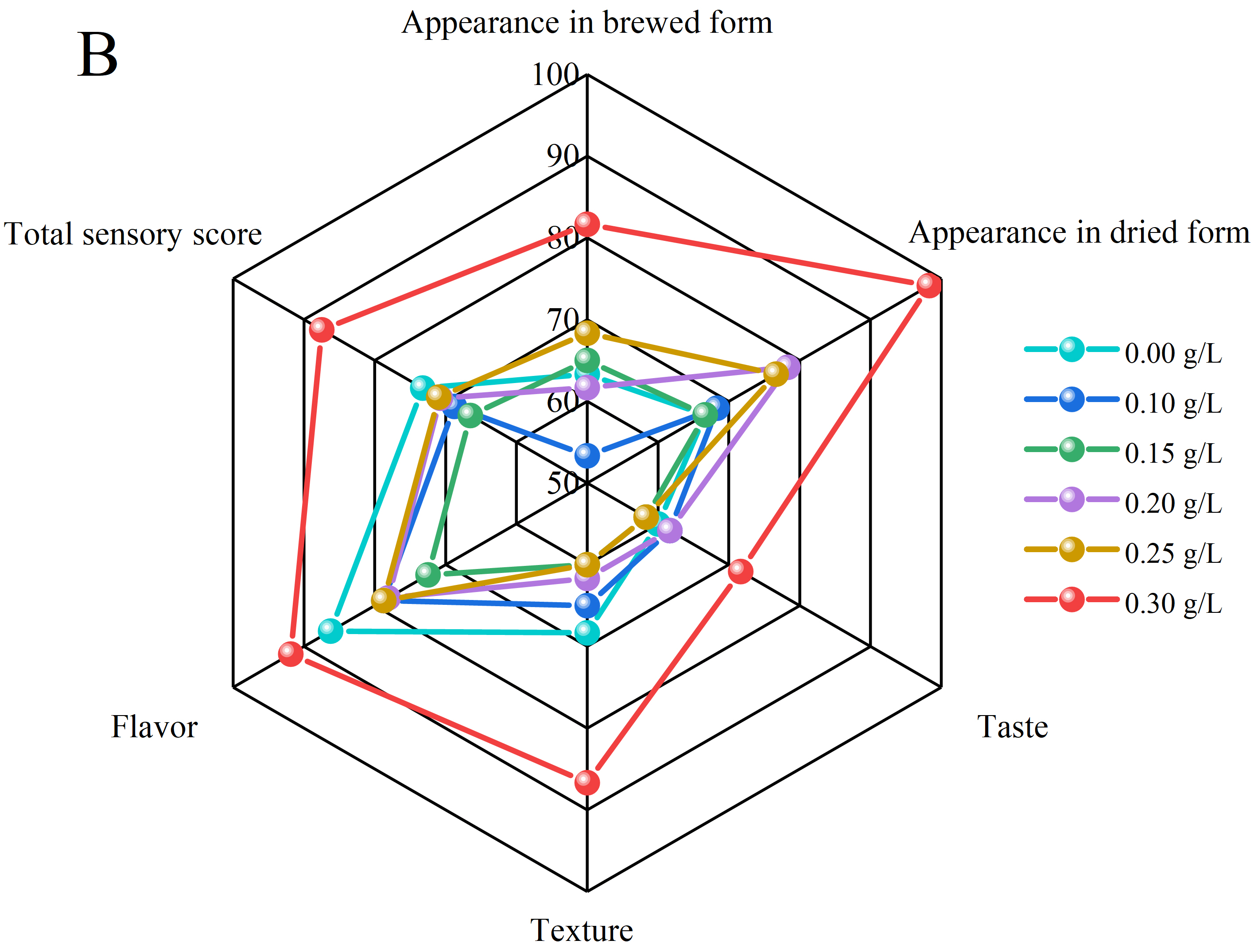


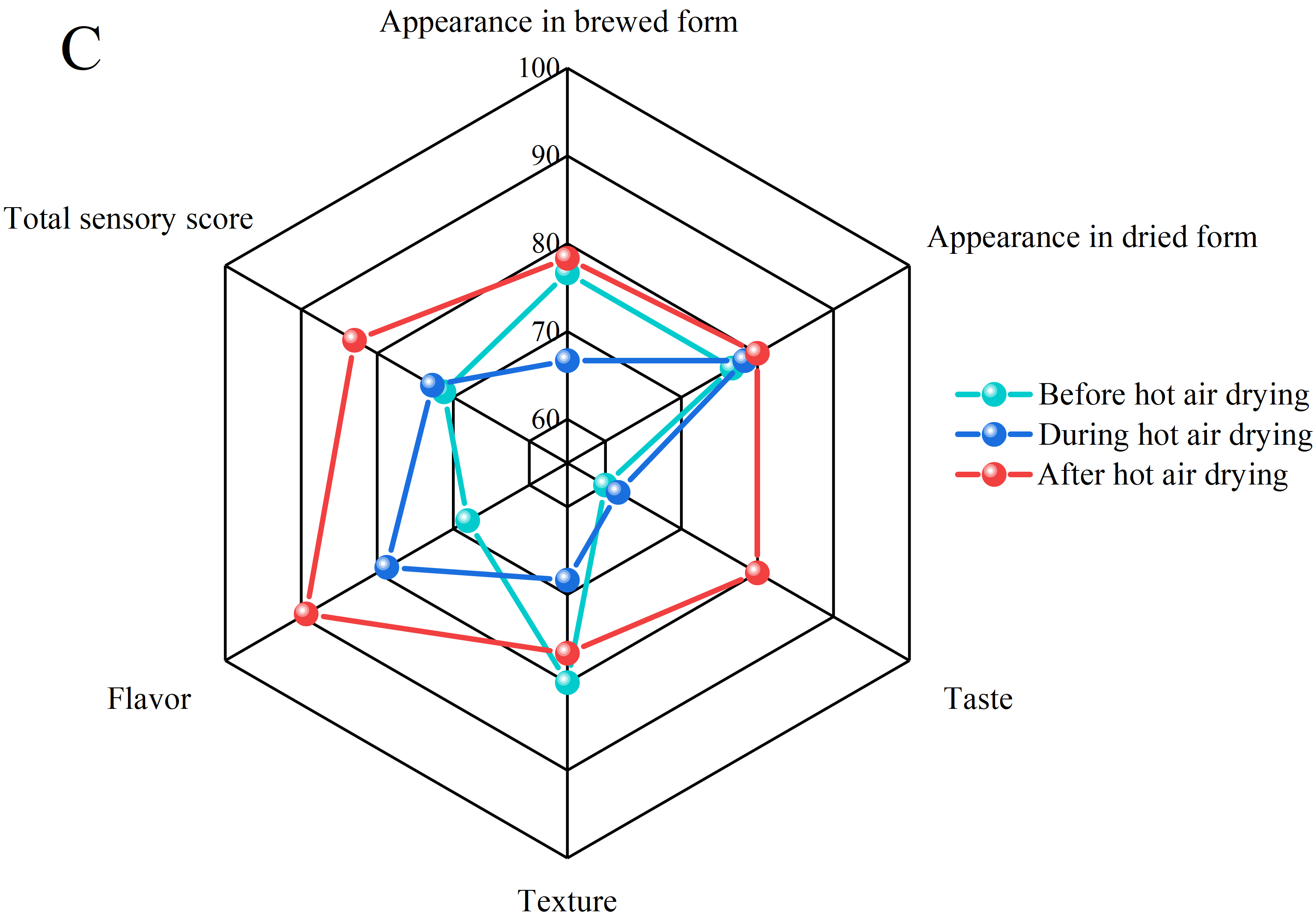


Fig.S1 Effects of amino acid type (A), concentration (B), addition timing (C) on the sensory scores of LRWPs.

| State | Category | Evaluation criteria | Score/ Grade | Weight |
| --- | --- | --- | --- | --- |
| In dried form | Appearance | Uniform color, smooth texture without noticeable lumps, bright luster | 67~100 | 0.15 |
|  |  | Relatively uniform color, slight lumpiness, and a dim luster | 34~66 |  |
|  |  | Uneven color, pronounced lumpiness, and a lackluster appearance | ＜33 |  |
| In brewed form | Appearance | Easily forms a smooth and gelatinous texture | 67~100 | 0.15 |
|  |  | Forms a relatively gelatinous texture with slight smoothness | 34~66 |  |
|  |  | Barely forms a gelatinous texture and lacks smoothness | ＜33 |  |
|  | Flavor | Prominent baking aroma with a strong association to the natural scent of lotus root | 67~100 | 0.4 |
|  |  | Mild baking aroma with a noticeable association to the scent of lotus root | 34~66 |  |
|  |  | No distinct baking aroma, but the scent remains associated with lotus root | ＜33 |  |
|  | Taste | Smooth in mouthfeel, no particulate | 67~100 | 0.15 |
|  |  | Relatively smooth mouthfeel with slight particulate presence | 34~66 |  |
|  |  | Rough mouthfeel with a strong presence of particulates | ＜33 |  |
|  | Texture | Consistent texture with a tight structure and uniform composition | 67~100 | 0.15 |
|  |  | Relatively consistent texture with a slightly tight structure and uniform composition | 34~66 |  |
|  |  | Lacking consistency, with no tight structure and uneven composition | ＜33 |  |

**Table S1.** Sensory evaluation criteria of LRWP.

**Table S2**. Volatile compounds at different processing stages.

| No. | Compound | Molecular formula | Retention index | Migration time | LRWP-Ⅰ | LRWP-Ⅱ | LRWP-Ⅲ | LRWP-Ⅳ |
| --- | --- | --- | --- | --- | --- | --- | --- | --- |
|  | **Alcohols** |  |  |  |  |  |  |  |
| 6 | 2-octanol monomer | C_8_H_18_O | 717.16 | 1.4605 | + | + | + | + |
| 15 | 2-furanmethanol | C_5_H_6_O_2_ | 377.028 | 1.3709 | + | + | + | + |
| 16 | 3-heptanol | C_7_H_16_O | 385.621 | 1.3274 | + | + | + | + |
| 25 | pentan-1-ol | C_5_H_12_O | 226.95 | 1.2553 |  | + | + | + |
| 28 | Isopulegol | C_10_H_18_O | 823.387 | 1.8931 | + | + | + | + |
| 37 | beta-citronellol | C_10_H_20_O | 986.032 | 1.8318 | + | + | + | + |
| 38 | 4-methyl-2-pentanol | C_6_H_14_O | 238.177 | 1.5517 |  |  | + | + |
| 60 | 3-methyl-3-buten-1-ol | C_5_H_10_O | 201.661 | 1.2468 |  | + | + | + |
| 62 | 2-phenylethanol | C_8_H_10_O | 831.639 | 1.5166 |  |  | + | + |
| 64 | (*Z*)-3-octen-1-ol dimer | C_8_H_16_O | 659.399 | 1.7499 | + | + | + | + |
| 76 | 2-ethyl-1-hexanol | C_8_H_18_O | 634.165 | 1.4076 |  | + | + | + |
| 78 | (*Z*)-3-octen-1-ol monomer | C_8_H_16_O | 660.503 | 1.7408 |  |  | + | + |
| 79 | 2-octanol dimer | C_8_H_18_O | 588.104 | 1.439 |  |  | + | + |
| 86 | (*Z*)-6-nonen-1-ol | C_9_H_18_O | 905.532 | 1.7606 |  |  |  | + |
| 92 | 1-heptanol | C_7_H_16_O | 562.858 | 1.7461 |  |  |  | + |
| 98 | linalool oxide | C_10_H_18_O_2_ | 833.671 | 1.8131 |  |  |  | + |
|  | **Aldehydes** |  |  |  |  |  |  |  |
| 41 | benzene acetaldehyde | C_8_H_8_O | 617.561 | 1.2537 |  | + | + | + |
| 47 | heptanal | C_7_H_14_O | 276.641 | 1.6939 | + | + | + | + |
| 54 | (*E*)- 2-heptenal | C_7_H_12_O | 521.29 | 1.2445 | + | + | + | + |
| 57 | (*E*)-2-octenal | C_8_H_14_O | 767.446 | 1.33 | + | + | + | + |
| 61 | citronellal | C_10_H_18_O | 990.173 | 1.8963 |  |  | + | + |
|  | **Acids** |  |  |  |  |  |  |  |
| 2 | nonanoic acid | C_9_H_18_O_2_ | 1047.77 | 1.5561 | + |  | + | + |
| 13 | allylacetic acid | C_5_H_8_O_2_ | 415.296 | 1.4414 | + |  | + | + |
| 35 | 2-methylbutanoic acid monomer | C_5_H_10_O_2_ | 374.696 | 1.4773 | + | + | + | + |
| 42 | 3-methyl valeric acid | C_6_H_12_O_2_ | 575.886 | 1.2806 |  | + | + | + |
| 50 | 2-methylbutanoic acid dimer | C_5_H_10_O_2_ | 317.327 | 1.4806 | + | + | + | + |
| 58 | 2-heptenoic acid | C_7_H_12_O_2_ | 966.164 | 1.4167 |  |  | + | + |
| 96 | 2-methylpropanoic acid | C_4_H_8_O_2_ | 245.153 | 1.2274 |  | + | + | + |
|  | **Esters** |  |  |  |  |  |  |  |
| 4 | furfuryl propionate | C_8_H_10_O_3_ | 835.936 | 1.4843 | + | + | + | + |
| 5 | acetic acid, hexyl ester | C_8_H_16_O_2_ | 723.493 | 1.4177 | + | + | + | + |
| 10 | isopentyl propanoate | C_8_H_16_O_2_ | 649.188 | 1.8322 | + | + | + | + |
| 17 | propyl propanoate monomer | C_6_H_12_O_2_ | 269.033 | 1.5857 | + |  | + | + |
| 18 | isopropyl methylphosphonofluoridate | C_4_H_10_FO_2_P | 272.225 | 1.4736 | + | + | + | + |
|  |  |  |  |  |  |  |  |  |
| 19 | acetic acid, 2-methylpropyl ester | C_6_H_12_O_2_ | 177.007 | 1.5994 | + | + | + | + |
| 20 | methyl 2-methylbutanoate | C_6_H_12_O_2_ | 177.53 | 1.544 | + | + | + | + |
| 33 | methyl heptanoate | C_8_H_16_O_2_ | 653.398 | 1.7995 | + | + | + | + |
| 34 | isoamyl butyrate | C_9_H_18_O_2_ | 657.763 | 1.9041 |  |  | + | + |
| 36 | 2-methylpropyl butanoate | C_8_H_16_O_2_ | 454.903 | 1.3301 | + | + | + | + |
| 43 | ethyl 3-methylbutanoate | C_7_H_14_O_2_ | 291.896 | 1.2524 |  | + | + | + |
| 46 | butyl butanoate monomer | C_8_H_16_O_2_ | 634.212 | 1.3381 |  | + | + | + |
| 48 | isopropyl 2-methylbutanoate | C_8_H_16_O_2_ | 271.005 | 1.7573 | + | + | + | + |
| 52 | butyl butanoate dimer | C_8_H_16_O_2_ | 508.858 | 1.3305 | + | + | + | + |
| 55 | butanoic acid 3-methylethyl ester | C_7_H_14_O_2_ | 443.93 | 1.2541 | + | + | + | + |
| 63 | propanedioic acid, diethyl ester | C_7_H_12_O_4_ | 717.131 | 1.2472 |  | + | + | + |
| 67 | butanoic acid, 3-methylbutyl ester | C_9_H_18_O_2_ | 585.04 | 1.38 |  |  | + | + |
| 73 | butyl pentanoate monomer | C_9_H_18_O_2_ | 828.599 | 1.9396 | + |  | + | + |
| 74 | butyl formate | C_5_H_10_O_2_ | 124.103 | 1.2122 | + |  |  |  |
| 80 | isobutyl isovalerate | C_9_H_18_O_2_ | 564.523 | 1.886 |  |  |  | + |
| 83 | propyl propanoate dimer | C_6_H_12_O_2_ | 188.162 | 1.2138 |  | + | + | + |
| 84 | 2-methylpropyl butanoate | C_8_H_16_O_2_ | 405.672 | 1.3333 | + | + | + | + |
| 87 | ethyl 3-(methylthio)propanoate | C_6_H_12_O_2_S | 831.31 | 1.6792 |  |  | + | + |
| 89 | butyl pentanoate dimer | C_9_H_18_O_2_ | 833.325 | 1.9337 |  |  | + | + |
|  | **Ketones** |  |  |  |  |  |  |  |
| 12 | 5-methyl-2-hepten-4-one | C_8_H_14_O | 560.819 | 1.6939 |  |  | + | + |
| 14 | cyclohexen-2-one | C_6_H_8_O | 376.019 | 1.4067 | + |  |  |  |
| 27 | n-ethylpyrrolidone | C_6_H_11_NO | 958.832 | 1.5584 | + | + | + | + |
| 32 | 1-octen-3-one monomer | C_8_H_14_O | 659.473 | 1.682 | + | + | + | + |
| 44 | cyclopentanone | C_5_H_8_O | 195.708 | 1.3298 |  | + | + | + |
| 71 | 1-penten-3-one | C_5_H_8_O | 83.774 | 1.318 | + | + | + | + |
| 72 | abhexone dimer | C_7_H_10_O_3_ | 888.245 | 1.4859 | + | + | + | + |
|  |  |  |  |  |  |  |  |  |
| 81 | 1-octen-3-one dimer | C_8_H_14_O | 500.717 | 1.676 |  |  |  | + |
| 88 | abhexone monomer | C_7_H_10_O_3_ | 986.47 | 1.8149 | + | + | + | + |
| 94 | 3-hepten-2-one | C_7_H_12_O | 567.906 | 1.6355 |  |  | + | + |
| 97 | levo-carvone | C_10_H_14_O | 910.339 | 1.8147 |  |  |  | + |
| 99 | 1-octen-3-one polymer | C_8_H_14_O | 461.552 | 1.6812 |  |  |  | + |
|  | **Alkenes** |  |  |  |  |  |  |  |
| 9 | *α*-phellandrene | C_10_H_16_ | 673.701 | 1.69 | + | + | + | + |
| 11 | *β*-myrcene | C_10_H_16_ | 560.819 | 1.6407 |  |  | + | + |
| 65 | *δ*-3-carene polymer | C_10_H_16_ | 590.215 | 1.6804 |  |  | + | + |
| 66 | *β*-ocimene | C_10_H_16_ | 755.792 | 1.6803 |  |  | + | + |
| 68 | camphene | C_10_H_16_ | 425.735 | 1.2114 |  |  | + | + |
| 77 | *δ*-3-carene dimer | C_10_H_16_ | 661.5 | 1.6758 | + | + | + | + |
| 82 | *δ*-3-carene monomer | C_10_H_16_ | 700.643 | 1.6819 |  |  | + | + |
| 90 | *β*-pinene | C_10_H_16_ | 459.74 | 1.2176 |  |  | + | + |
|  | **Pyrazines** |  |  |  |  |  |  |  |
| 1 | 2-isobutyl-3-methoxypyrazine | C_9_H_14_N_2_O | 997.556 | 1.8382 | + | + | + | + |
| 51 | 2,3,5-trimethylpyrazine | C_7_H_10_N_2_ | 721.684 | 1.62 | + | + | + | + |
| 56 | 2-methylpyrazine dimer | C_5_H_6_N_2_ | 223.937 | 1.3948 |  | + | + | + |
| 93 | 2-ethylpyrazine | C_6_H_8_N_2_ | 414.311 | 1.5249 |  |  | + | + |
| 95 | 2-methylpyrazine monomer | C_5_H_6_N_2_ | 239.853 | 1.3959 |  | + | + | + |
|  | **Miscellaneous compounds** |  |  |  |  |  |  |  |
| 3 | veratrole | C_8_H_10_O_2_ | 829.512 | 1.5784 | + |  |  |  |
| 7 | 2-acetylpyrrole | C_6_H_7_NO | 720.356 | 1.4947 | + |  |  |  |
| 8 | triethylenediamine | C_6_H_12_N_2_ | 632.378 | 1.5133 | + |  |  |  |
| 22 | 1,3-dioxolane, 2,4-dimethyl, cis | C_5_H_10_O_2_ | 104.214 | 1.3922 | + | + | + | + |
| 23 | 1,4-dioxan monomer | C_4_H_8_O_2_ | 180.063 | 1.3191 | + | + | + | + |
| 24 | 1,4-dioxan dimer | C_4_H_8_O_2_ | 92.505 | 1.323 |  | + | + | + |
| 26 | thiazole | C_3_H_3_NS | 118.776 | 1.2581 | + | + | + | + |
| 39 | 2,2,4,6,6-pentamethyl-heptane | C_12_H_26_ | 512.575 | 1.3762 |  | + | + | + |
| 40 | decalin | C_10_H_18_ | 699.399 | 1.2722 |  | + | + | + |
| 45 | pyrrolidine | C_4_H_9_N | 70.309 | 1.2715 |  |  | + | + |
| 49 | n-nitrosodiethylamine | C_4_H_10_N_2_O | 419.157 | 1.5292 |  |  | + | + |
| 69 | 2-butoxyethanol | C_6_H_14_O_2_ | 430.854 | 1.3042 |  |  | + | + |
| 75 | triethylamine | C_6_H_15_N | 69.68 | 1.22 | + | + |  |  |
| 91 | diethyl disulfide | C_4_H_10_S_2_ | 522.116 | 1.2821 |  | + | + | + |
|  | **Unidentified** |  |  |  |  |  |  |  |
| 21 | unidentified 1 | - | - | - |  | + | + | + |
| 29 | unidentified 2 | - | - | - |  |  | + | + |
| 30 | unidentified 3 | - | - | - |  |  | + | + |
| 31 | unidentified 4 | - | - | - | + | + | + | + |
| 53 | unidentified 5 | - | - | - | + | + | + | + |
| 59 | unidentified 6 | - | - | - |  |  | + | + |
| 70 | unidentified 7 | - | - | - |  | + | + | + |
| 85 | unidentified 8 | - | - | - |  | + | + | + |

Notes: “+” indicates that the compound was detected, “-” means compound unknown.

**Table S3**. Differential feature metabolites at different processing stages.

| Superclasses | RT | Molecular Weight | Formula | Name |
| --- | --- | --- | --- | --- |
| Alkaloids (9) | 1.27 | 215.1389 | C_10_H_18_N_2_O_3_ | Valylproline |
|  | 0.881 | 163.1229 | C_10_H_14_N_2_ | Nicotine |
|  | 2.737 | 175.12304 | C_11_H_14_N_2_ | N-Methyltryptamine |
|  | 4.342 | 286.14369 | C_17_H_19_NO_3_ | Morphine |
|  | 5.317 | 300.15931 | C_18_H_21_NO_3_ | Codeine |
|  | 7.135 | 625.32706 | C_38_H_44_N_2_O_6_ | Neferine |
|  | 8.581 | 296.16441 | C_19_H_21_NO_2_ | Nuciferine |
|  | 13.504 | 306.07596 | C_18_H_11_NO_4_ | Atherospermidine |
|  | 6.321 | 314.17481 | C_19_H_23_NO_3_ | Ethylmorphine |
| Amino acid related compounds (9) | 0.913 | 293.09871 | C_10_H_18_N_2_O_8_ | N-Glycosyl-L-asparagine |
|  | 0.807 | 217.11928 | C_9_H_18_N_2_O_4_ | D-Lysopine |
|  | 0.807 | 307.15104 | C_12_H_24_N_2_O_7_ | Psicoselysine |
|  | 0.914 | 128.03536 | C_5_H_7_NO_3_ | 4-Oxoproline |
|  | 1.195 | 130.04988 | C_5_H_7_NO_3_ | L-Pyroglutamic acid |
|  | 1.286 | 216.08673 | C_9_H_13_NO_5_ | Succinylproline |
|  | 0.76 | 147.11291 | C_6_H_11_NO_2_ | Pipecolic acid |
|  | 5.215 | 210.07711 | C_10_H_13_NO_4_ | 2-Amino-3-(4-hydroxy-3-methoxyphenyl) propanoic acid |
|  | 1.282 | 406.09091 | C_14_H_21_N_3_O_9_S | S-Succinylglutathione |
| Amino acids (6) | 0.766 | 147.11276 | C_6_H_14_N_2_O_2_ | DL-Lysine |
|  | 1.28 | 147.11266 | C_6_H_14_N_2_O_2_ | L-Lysine |
|  | 0.832 | 146.04589 | C_5_H_9_NO_4_ | L-Glutamic acid |
|  | 0.835 | 145.0619 | C_5_H_10_N_2_O_3_ | D-(-)-Glutamine |
|  | 0.826 | 147.07639 | C_5_H_10_N_2_O_3_ | DL-Glutamine |
|  | 1.255 | 130.08742 | C_6_H_13_NO_2_ | DL-beta-Leucine |
| Benzenoids (18) | 0.767 | 413.15494 | C_27_H_21_FO_3_ | 1-(4-fluorophenyl)-2-(4-methoxyphenyl)-4-(2-naphthyl)butane-1,4-dione |
|  | 0.901 | 296.11671 | C_14_H_18_ClN_3_O_2_ | Triadimenol |
|  | 1.333 | 154.0499 | C_7_H_7_NO_3_ | Mesalamine |
|  | 2.385 | 237.12335 | C_15_H_20_O_2_ | (4aR,5R,6R)-6-hydroxy-4a,5-dimethyl-3-(prop-1-en-2-yl)-2,4a,5,6,7,8-hexahydronaphthalen-2-one |
|  | 2.836 | 253.11916 | C_12_H_18_N_2_O_4_ | Midodrine |
|  | 3.587 | 159.09172 | C_10_H_10_N_2_ | 1,5-Naphthalenediamine |
|  | 3.588 | 144.08084 | C_10_H_9_N | 2-Naphthylamine |
|  | 4.531 | 443.19221 | C_22_H_28_N_4_O_6_ | Mitoxantrone |
|  | 5.019 | 163.04021 | C_9_H_8_O_3_ | Phenylpyruvic acid |
|  | 5.197 | 121.02956 | C_7_H_6_O_2_ | Benzoic acid |
|  | 5.535 | 374.15981 | C_20_H_20_O_6_ | (2*Z*)-1,4-bis(4-hydroxy-3-methoxyphenyl)-2,3-dimethylbut-2-ene-1,4-dione |
|  | 6.289 | 119.05025 | C_8_H_8_O | Phenylacetaldehyde |
|  | 7.074 | 219.08044 | C_16_H_10_O | 1-Pyrenol |
|  | 7.439 | 179.0703 | C_10_H_10_O_3_ | Ferulaldehyde |
|  | 7.482 | 137.02448 | C_7_H_6_O_3_ | Salicylic acid |
|  | 9.011 | 133.10117 | C_10_H_12_ | Tetralin |
|  | 1.29 | 239.06724 | C_10_H_12_N_2_O_5_ | Dinoterb |
|  | 0.848 | 325.12515 | C_20_H_19_FO_3_ | 2,2,4-Trimethyl-3-(4-fluorophenyl)-2H-1-benzopyran-7-ol acetate |
| Carbohydrates (21) | 0.769 | 471.21837 | C_21_H_36_O_10_ | (2R,3S,4S,5R,6R)-2-({[(2R,3R,4R,5S)-3,4-dihydroxy-5-(hydroxymethyl)oxolan-2-yl]oxy}methyl)-6-{[(2*E*)-3,7-dimethylocta-2,6-dien-1-yl]oxy}oxane-3,4,5-triol |
|  | 0.799 | 421.07543 | C_19_H_18_O_11_ | 1,5,8-Trihydroxy-9-oxo-9H-xanthen-3-yl beta-D-glucopyranoside |
|  | 0.824 | 455.11591 | C_18_H_24_O_12_ | Asperulosidic acid |
|  | 0.839 | 365.10519 | C_12_H_22_O_11_ | D-(+)-Maltose |
|  | 0.85 | 181.07175 | C_6_H_14_O_6_ | D-(-)-Mannitol |
|  | 0.857 | 446.18643 | C_23_H_28_ClN_3_O_4_ | 2,5-Anhydro-6-[(2-chlorobenzoyl)amino]-4,6-dideoxy-4-(4-phenyl-1-piperazinyl)-D-galactitol |
|  | 0.877 | 296.13378 | C_11_H_18_O_8_ | 4-(beta-D-Glucopyranosyloxy)-2-methylenebutanoic acid |
|  | 0.879 | 337.1602 | C_16_H_26_O_6_ | (2R,3R,4S,5S,6R)-2-{[(2*E*)-4-ethenyl-2,5-dimethylhexa-2,5-dien-1-yl]oxy}-6-(hydroxymethyl)oxane-3,4,5-triol |
|  | 0.894 | 310.11301 | C_11_H_19_NO_9_ | N-Acetylneuraminic acid |
|  | 0.906 | 337.07736 | C_12_H_18_O_11_ | L-Ascorbic acid-2-glucoside |
|  | 1.195 | 313.11415 | C_11_H_20_O_10_ | Vicianose |
|  | 1.223 | 434.20175 | C_19_H_28_O_10_ | (2R,3R,4R,5R,6S)-2-{[(2R,3S,4S,5R,6R)-6-(benzyloxy)-3,4,5-trihydroxyoxan-2-yl]methoxy}-6-methyloxane-3,4,5-triol |
|  | 5.368 | 431.19232 | C_19_H_30_O_8_ | 3-Hydroxy-3,5,5-trimethyl-4-(3-oxo-1-buten-1-ylidene)cyclohexyl beta-D-glucopyranoside |
|  | 5.402 | 464.21285 | C_20_H_30_O_11_ | (2R,3R,4S,5S,6R)-2-[2-(4-methoxyphenyl)ethoxy]-6-({[(2S,3R,4S,5S)-3,4,5-trihydroxyoxan-2-yl]oxy}methyl)oxane-3,4,5-triol |
|  | 5.57 | 295.13974 | C_11_H_22_O_6_ | 2-Methylbutyl beta-D-glucopyranoside |
|  | 6.301 | 552.31413 | C_29_H_42_O_9_ | (3beta,9xi)-3-(beta-D-Glucopyranosyloxy)-14-hydroxycarda-5,20(22)-dienolide |
|  | 6.624 | 643.32964 | C_32_H_50_O_13_ | Steviolbioside |
|  | 6.87 | 461.14433 | C_23_H_24_O_10_ | Cladrin 7-O-glucoside |
|  | 7.171 | 435.12968 | C_21_H_24_O_10_ | Trilobatin |
|  | 7.689 | 433.11397 | C_21_H_22_O_10_ | 7-Hydroxy-2-(4-hydroxyphenyl)-4-oxo-3,4-dihydro-2H-chromen-5-yl beta-D-glucopyranoside |
|  | 8.118 | 579.17258 | C_27_H_32_O_14_ | 7-{[4,5-dihydroxy-6-(hydroxymethyl)-3-{[(2S,3R,4R,5R,6S)-3,4,5-trihydroxy-6-methyloxan-2-yl]oxy}oxan-2-yl]oxy}-5-hydroxy-2-(4-hydroxyphenyl)-3,4-dihydro-2H-1-benzopyran-4-one |
| Fatty acyls (17) | 5.325 | 245.18598 | C_12_H_24_N_2_O_3_ | N-(6-Aminohexanoyl)-6-aminohexanoate |
|  | 14.388 | 295.22672 | C_18_H_30_O_3_ | 2(R)-HOT |
|  | 14.731 | 277.21623 | C_18_H_28_O_2_ | Stearidonic acid |
|  | 16.288 | 295.22674 | C_18_H_30_O_3_ | 9-Oxo-10(E),12(E)-octadecadienoic acid |
|  | 18.707 | 279.23123 | C_18_H_30_O_2_ | Alpha-Eleostearic acid |
|  | 18.999 | 271.22755 | C_16_H_32_O_3_ | 16-Hydroxyhexadecanoic acid |
|  | 19.403 | 300.28986 | C_18_H_37_NO_2_ | Palmitoyl ethanolamide |
|  | 14.075 | 295.22674 | C_18_H_30_O_3_ | 9-Oxo-ODE |
|  | 15.75 | 295.22763 | C_18_H_34_O_4_ | (+/-)9(10)-DiHOME |
|  | 16.349 | 293.21199 | C_18_H_32_O_4_ | (+/-)9-HpODE |
|  | 17.954 | 324.28958 | C_20_H_37_NO_2_ | Linoleoyl ethanolamide |
|  | 18.59 | 391.22542 | C_20_H_36_O_5_ | 8-iso Prostaglandin F1 |
|  | 21.035 | 293.24734 | C_19_H_32_O_2_ | 9(Z),11(E),13(E)-Octadecatrienoic Acid methyl ester |
|  | 21.088 | 283.26384 | C_18_H_34_O_2_ | Ethyl palmitoleate |
|  | 21.269 | 351.28928 | C_22_H_38_O_3_ | ethyl 5R,6R-epoxy-7-eicosynoate |
|  | 22 | 295.26299 | C_19_H_34_O_2_ | Methyl linoleate |
|  | 17.132 | 330.33642 | C_20_H_40_O_2_ | Arachidic Acid |
| Flavonoids (19) | 1.434 | 305.06567 | C_15_H_12_O_7_ | Taxifolin |
|  | 2.672 | 443.09733 | C_22_H_18_O_10_ | (2R,3R)-2-(3,4-dihydroxyphenyl)-5,7-dihydroxy-3,4-dihydro-2H-1-benzopyran-3-yl 3,4,5-trihydroxybenzoate |
|  | 2.675 | 223.02473 | C_10_H_8_O_6_ | Sideretin |
|  | 4.465 | 465.10292 | C_21_H_20_O_12_ | Myricitrin |
|  | 4.629 | 355.10233 | C_16_H_18_O_9_ | 7-methoxy-6-{[(2S,3R,4S,5S,6R)-3,4,5-trihydroxy-6-(hydroxymethyl)oxan-2-yl]oxy}-2H-chromen-2-one |
|  | 5.445 | 289.07062 | C_15_H_12_O_6_ | (-)-Fustin |
|  | 5.532 | 449.10887 | C_21_H_22_O_11_ | 3,5-Dihydroxy-2-(4-hydroxyphenyl)-4-oxo-3,4-dihydro-2H-chromen-7-yl hexopyranoside |
|  | 6.298 | 479.0829 | C_21_H_20_O_13_ | Myricetin 3-O-beta-D-galactopyranoside |
|  | 7.705 | 275.09129 | C_15_H_14_O_5_ | Phloretin |
|  | 7.743 | 493.13403 | C_23_H_24_O_12_ | Tricin 5-O-beta-D-glucoside |
|  | 7.782 | 435.12964 | C_21_H_24_O_10_ | Phloridzin |
|  | 7.961 | 315.05094 | C_16_H_12_O_7_ | Isorhamnetin |
|  | 8.039 | 301.03524 | C_15_H_10_O_7_ | Quercetin |
|  | 8.65 | 287.0559 | C_15_H_12_O_6_ | Eriodictyol |
|  | 8.928 | 417.11896 | C_21_H_22_O_9_ | Isoliquiritin |
|  | 9.708 | 271.06088 | C_15_H_12_O_5_ | Naringenin |
|  | 9.845 | 271.06086 | C_15_H_12_O_5_ | Naringenin chalcone |
|  | 10.219 | 329.06656 | C_17_H_14_O_7_ | Jaceosidin |
|  | 12.159 | 255.06602 | C_15_H_12_O_4_ | Pinocembrin |
| Lipids (20) | 14.07 | 398.26662 | C_18_H_40_NO_6_P | Phytosphingosine 1-phosphate |
|  | 14.84 | 571.28943 | C_25_H_49_O_12_P | PI(16:0/0:0) |
|  | 14.859 | 640.34554 | C_29_H_54_NO_12_P | Am-LPE(18:2(9Z,12Z)0:0) |
|  | 15.172 | 597.30505 | C_27_H_51_O_12_P | PG-PG |
|  | 15.431 | 500.27469 | C_25_H_42_NO_7_P | PE(20:5(5Z,8Z,11Z,14Z,17Z)/0:0) |
|  | 15.432 | 478.29254 | C_23_H_44_NO_7_P | PE(18:2(9Z,12Z)/0:0) |
|  | 15.635 | 616.34528 | C_27_H_54_NO_12_P | Am-LPE(16:0/0:0) |
|  | 15.987 | 483.27247 | C_22_H_45_O_9_P | PG(16:0/0:0) |
|  | 16.106 | 433.23584 | C_21_H_39_O_7_P | Oleoylglycerone phosphate |
|  | 16.347 | 454.29263 | C_21_H_44_NO_7_P | Glycerophospho-N-palmitoyl ethanolamine |
|  | 16.694 | 480.30837 | C_23_H_46_NO_7_P | PE(0:0/18:1(9Z)) |
|  | 16.755 | 478.2936 | C_23_H_46_NO_7_P | PE(18:1(9Z)/0:0) |
|  | 17.116 | 393.23985 | C_19_H_37_O_6_P | CPA(16:0) |
|  | 17.423 | 419.25561 | C_21_H_39_O_6_P | CPA(18:1(9Z)) |
|  | 17.751 | 496.33947 | C_24_H_50_NO_7_P | 1-(2-methoxy-6*Z*-octadecenyl)-sn-glycero-3-phosphoethanolamine |
|  | 17.824 | 540.32977 | C_25_H_52_NO_9_P | 1-(2-methoxy-octadecanyl)-sn-glycero-3-phosphoserine |
|  | 18.171 | 566.34695 | C_27_H_54_NO_9_P | PS(21:0/0:0) |
|  | 17.185 | 409.23595 | C_19_H_39_O_7_P | 1-Palmitoylglycerol 3-phosphate |
|  | 19.861 | 279.23267 | C_18_H_32_O_2_ | Linoleic acid |
|  | 20.126 | 339.28921 | C_21_H_38_O_3_ | Glycidyl oleate |
| Lipids and lipid - like molecules (11) | 15.873 | 677.3715 | C_33_H_56_O_14_ | Gingerglycolipid A |
|  | 21.502 | 341.30478 | C_21_H_40_O_3_ | Glycidyl stearate |
|  | 0.797 | 333.05929 | C_9_H_19_O_11_P | Sn-Glycero-3-phospho-1-inositol |
|  | 10.103 | 327.21755 | C_18_H_32_O_5_ | Corchorifatty acid F |
|  | 15.81 | 337.27353 | C_21_H_38_O_4_ | 2-Linoleoyl glycerol |
|  | 17.48 | 435.25157 | C_21_H_41_O_7_P | Oleoyl-L-alpha-lysophosphatidic acid |
|  | 19.007 | 317.26862 | C_18_H_36_O_4_ | 9,10-Dihydroxystearate |
|  | 19.8 | 281.24734 | C_18_H_32_O_2_ | Linoelaidic acid |
|  | 18.759 | 355.28389 | C_21_H_38_O_4_ | 1-Linoleoyl glycerol |
|  | 20.022 | 331.28398 | C_19_H_38_O_4_ | 1-Palmitoylglycerol |
|  | 20.127 | 357.29955 | C_21_H_40_O_4_ | Monoolein |
| Nucleic acids and related compounds (7) | 0.882 | 244.09268 | C_9_H_13_N_3_O_5_ | Cytidine |
|  | 0.903 | 211.07126 | C_9_H_12_N_2_O_5_ | 2'-Deoxyuridine |
|  | 1.194 | 330.05933 | C_10_H_12_N_5_O_6_P | Adenosine 3'5'-cyclic monophosphate |
|  | 1.2 | 136.06177 | C_5_H_5_N_5_ | Adenine |
|  | 1.215 | 152.05659 | C_5_H_5_N_5_O | Guanine; 2-Amino-6-hydroxypurine |
|  | 1.219 | 268.10371 | C_10_H_13_N_5_O_4_ | Adenosine |
|  | 1.222 | 284.09864 | C_10_H_13_N_5_O_5_ | Guanosine |
| Organic acids and derivatives (36) | 9.174 | 131.04917 | C_9_H_8_O_2_ | Cinnamic acid |
|  | 19.313 | 161.05978 | C_10_H_10_O_3_ | 3-Methoxycinnamic acid |
|  | 0.909 | 173.00911 | C_6_H_6_O_6_ | Trans-Aconitic acid |
|  | 0.826 | 189.0048 | C_6_H_6_O_7_ | Oxalosuccinate |
|  | 0.844 | 215.03263 | C_5_H_13_O_7_P | 2-C-Methyl-D-erythritol 4-phosphate |
|  | 0.872 | 195.05086 | C_6_H_12_O_7_ | Galactonic acid |
|  | 0.881 | 206.10225 | C_8_H_12_O_5_ | Methyl 3,4,5-trihydroxycyclohex-1-ene-1-carboxylate |
|  | 0.881 | 291.1185 | C_11_H_18_N_2_O_7_ | N-Succinyl-LL-2,6-diaminoheptanedioate |
|  | 0.9 | 277.10286 | C_10_H_16_N_2_O_7_ | Gamma-L-Glutamyl-L-glutamic acid |
|  | 0.924 | 115.00365 | C_4_H_4_O_4_ | Fumaric acid |
|  | 0.933 | 457.11945 | C_21_H_22_N_4_O_6_S | Tomudex |
|  | 1.192 | 146.09235 | C_5_H_11_N_3_O_2_ | 4-Guanidinobutyric acid |
|  | 1.195 | 290.13442 | C_11_H_19_N_3_O_6_ | Ophthalmate |
|  | 1.197 | 613.15906 | C_20_H_32_N_6_O_12_S_2_ | L-Glutathione oxidized |
|  | 1.226 | 181.06412 | C_5_H_12_N_2_O_3_S | L-Methionine sulfoximine |
|  | 1.232 | 172.0981 | C_8_H_15_NO_3_ | N-Acetyl-L-leucine |
|  | 1.252 | 202.10846 | C_9_H_17_NO_4_ | 3-[(methoxycarbonyl)amino]-2,2,3-trimethylbutanoic acid |
|  | 1.319 | 205.0353 | C_7_H_10_O_7_ | 3-Hydroxy-3-(methoxycarbonyl)pentanedioic acid |
|  | 1.376 | 294.15457 | C_12_H_20_O_7_ | Citroflex 2 |
|  | 1.399 | 236.09267 | C_12_H_15_NO_4_ | 5-Amino-3-(4-methoxyphenyl)-5-oxopentanoic acid |
|  | 3.621 | 129.0558 | C_6_H_10_O_3_ | 2-Hydroxyethyl methacrylate |
|  | 4.407 | 295.12877 | C_14_H_18_N_2_O_5_ | Aspartame |
|  | 7.882 | 191.03389 | C_10_H_6_O_4_ | 2-oxo-2H-chromene-3-carboxylic acid |
|  | 10.03 | 351.21388 | C_18_H_32_O_5_ | (11E,15Z)-9,10,13-trihydroxyoctadeca-11,15-dienoic acid |
|  | 14.835 | 423.274 | C_24_H_38_O_6_ | Lovastatin acid |
|  | 15.268 | 333.20348 | C_20_H_30_O_5_ | 5-(4-carboxy-3-methylbutyl)-5,6,8a-trimethyl-3-oxo-3,4,4a,5,6,7,8,8a-octahydronaphthalene-1-carboxylic acid |
|  | 15.322 | 391.24536 | C_23_H_36_O_6_ | (3R,5R)-7-[(1S,2S,8S,8aR)-2-methyl-8-[(2-methylbutanoyl)oxy]-1,2,6,7,8,8a-hexahydronaphthalen-1-yl]-3,5-dihydroxyheptanoic acid |
|  | 17.014 | 417.23995 | C_23_H_32_N_2_O_5_ | Ramipril |
|  | 0.93 | 439.08529 | C_18_H_34_Br_2_O_2_ | 9,10-dibromo-stearic acid |
|  | 10.031 | 293.21085 | C_18_H_28_O_3_ | 12-oxo Phytodienoic Acid |
|  | 10.619 | 295.22675 | C_18_H_30_O_3_ | 13(S)-HOTrE |
|  | 10.62 | 353.22969 | C_18_H_34_O_5_ | (12*Z*)-9,10,11-trihydroxyoctadec-12-enoic acid |
|  | 10.69 | 329.23317 | C_18_H_34_O_5_ | (15*Z*)-9,12,13-Trihydroxy-15-octadecenoic acid |
|  | 14.86 | 309.20701 | C_18_H_30_O_4_ | 9-Hydroxy-12-oxo-10(*E*),15(*Z*)-octadecadienoic acid |
|  | 19.674 | 313.27351 | C_19_H_36_O_3_ | 3-oxo-nonadecanoic acid |
|  | 0.908 | 111.00873 | C_5_H_6_O_4_ | Mesaconic acid |
| Organic nitrogen compound (21) | 0.887 | 192.08657 | C_7_H_13_NO_5_ | 2-Amino-3,7-dideoxy-D-threo-hept-6-ulosonic acid |
|  | 1.202 | 217.11823 | C_9_H_16_N_2_O_4_ | gamma-Glutamyl-gamma-aminobutyraldehyde |
|  | 0.885 | 342.16572 | C_19_H_23_N_3_OS | N1-(3,4-dimethylphenyl)-2-[(2-propoxyphenyl)methylene]-1-hydrazinecarbothioamide |
|  | 0.762 | 429.10248 | C_17_H_15_F_3_N_4_O_6_ | (5-nitro-2-furyl){4-[2-nitro-4-(trifluoromethyl)phenyl]-1,4-diazepan-1-yl}methanone |
|  | 0.773 | 146.1652 | C_7_H_19_N_3_ | Spermidine |
|  | 0.819 | 290.06354 | C_8_H_12_N_5_O_5_P | PMEG |
|  | 0.821 | 262.06863 | C_14_H_12_FNOS | 2-Fluoro-N-[3-(methylthio)phenyl]benzamide |
|  | 0.825 | 347.08498 | C_10_H_15_N_6_O_6_P | 3'-Amino-3'-deoxy-AMP |
|  | 0.871 | 447.13509 | C_16_H_24_N_4_O_11_ | Staphyloferrin B |
|  | 1.197 | 164.07386 | C_6_H_13_NO_2_S | Ethionine |
|  | 1.226 | 304.14999 | C_12_H_21_N_3_O_6_ | Nicotianamine |
|  | 1.261 | 306.16572 | C_12_H_23_N_3_O_6_ | 6'-Oxolividamine |
|  | 1.273 | 256.11775 | C_12_H_17_NO_5_ | N-D-Glucosylarylamine |
|  | 1.281 | 188.1281 | C_8_H_7_ClFNO | 2-(2-chloro-4-fluorophenyl)acetamide |
|  | 2.507 | 184.06154 | C_8_H_11_NO_4_ | N-(3-oxo-butanoyl)-homoserine lactone |
|  | 4.695 | 130.15911 | C_8_H_19_N | N,N-Diisopropylethylamine (DIPEA) |
|  | 6.281 | 444.17655 | C_23_H_29_N_3_O_2_S_2_ | Thiothixene |
|  | 14.564 | 298.27386 | C_18_H_35_NO_2_ | 2-Aminooctadec-4-yne-1,3-diol |
|  | 16.657 | 401.28969 | C_22_H_40_O_6_ | Ascr#27 |
|  | 19.466 | 326.3051 | C_20_H_39_NO_2_ | Oleoyl ethanolamide |
|  | 0.884 | 216.12312 | C_10_H_17_NO_4_ | 2-Amino-9,10-epoxy-8-oxodecanoic acid |
| Organic oxygen compounds (27) | 0.784 | 338.98883 | C_6_H_14_O_12_P_2_ | Alpha-D-Glucose-1,6-bisphosphate |
|  | 0.799 | 259.02219 | C_6_H_13_O_9_P | Beta-D-Fructose 6-phosphate |
|  | 0.8 | 289.03288 | C_7_H_15_O_10_P | D-Sedoheptulose 7-phosphate |
|  | 0.873 | 105.01923 | C_3_H_6_O_4_ | (2R)-2,3-Dihydroxypropanoic acid |
|  | 0.942 | 117.01932 | C_4_H_8_O_5_ | L-Threonic acid |
|  | 1.19 | 246.1333 | C_11_H_16_O_5_ | 6-(3-hydroxybutan-2-yl)-5-(hydroxymethyl)-4-methoxy-2H-pyran-2-one |
|  | 1.194 | 217.08179 | C_8_H_12_N_2_O_5_ | 8-Hydroxyalanylclavam |
|  | 1.205 | 344.13366 | C_15_H_18_O_8_ | (2*E*)-3-(2-{[(2S,3R,4S,5S,6R)-3,4,5-trihydroxy-6-(hydroxymethyl)oxan-2-yl]oxy}phenyl)prop-2-enoic acid |
|  | 2.586 | 153.05581 | C_8_H_10_O_3_ | 3,4-Dihydroxyphenylethanol |
|  | 5.617 | 329.08775 | C_13_H_16_O_7_ | Helicid |
|  | 6.867 | 167.03505 | C_8_H_8_O_4_ | 2,4,6-Trihydroxyacetophenone |
|  | 7.213 | 540.24414 | C_26_H_34_O_11_ | 2-(hydroxymethyl)-6-{5-[3-(hydroxymethyl)-5-(3-hydroxypropyl)-7-methoxy-2,3-dihydro-1-benzofuran-2-yl]-2-methoxyphenoxy}oxane-3,4,5-triol |
|  | 7.977 | 377.15943 | C_20_H_24_O_7_ | 1,3-dihydroxy-1-(7-methoxy-2-oxo-2H-chromen-6-yl)-3-methylbutan-2-yl (2Z)-2-methylbut-2-enoate |
|  | 8.282 | 331.15375 | C_19_H_22_O_5_ | 7-(3,4-dihydroxyphenyl)-5-hydroxy-1-(4-hydroxyphenyl)heptan-3-one |
|  | 8.283 | 383.14641 | C_20_H_24_O_6_ | 2,5-bis(4-hydroxy-3-methoxyphenyl)-3,4-dimethyloxolan-3-ol |
|  | 8.605 | 551.26155 | C_30_H_40_O_8_ | 14-(acetyloxy)-6-(furan-3-yl)-12-hydroxy-1,7,11,15,15-pentamethyl-5-oxo-3-oxapentacyclo[8.8.0.0²,⁷.0¹¹,¹⁵.0¹⁴,¹⁸]octadecan-18-yl acetate |
|  | 8.684 | 153.09219 | C_9_H_16_O_3_ | 4-Hydroperoxy- |
|  | 8.851 | 441.11559 | C_21_H_22_O_9_ | 2-hydroxy-6-[(*E*)-2-phenylethenyl]-4-{[(2S,3R,4S,5S,6R)-3,4,5-trihydroxy-6-(hydroxymethyl)oxan-2-yl]oxy}benzoic acid |
|  | 9.18 | 341.13808 | C_20_H_22_O_6_ | 4-[(1S,3aR,4S,6aR)-4-(4-hydroxy-3-methoxyphenyl)-hexahydrofuro[3,4-c]furan-1-yl]-2-methoxyphenol |
|  | 10.44 | 183.13904 | C_11_H_20_O_2_ | Gamma-Undecalactone |
|  | 10.618 | 149.13255 | C_11_H_18_O | 2,5-undecadienal |
|  | 11.789 | 378.19103 | C_20_H_24_O_6_ | (3aR,4R,5R,11aR)-4-hydroxy-6,10-dimethyl-3-methylidene-2,8-dioxo-2H,3H,3aH,4H,5H,8H,11H,11aH-cyclodeca[b]furan-5-yl (2Z)-2-methylbut-2-enoate |
|  | 16.038 | 417.23989 | C_26_H_36_O_4_ | 5-[(10Z)-14-(3,5-dihydroxyphenyl)tetradec-10-en-1-yl]benzene-1,3-diol |
|  | 17.628 | 261.22126 | C_18_H_28_O | 6-[5]-ladderane-1-hexanol |
|  | 18.467 | 329.26853 | C_19_H_36_O_4_ | 1,4-dihydroxyheptadec-16-en-2-yl acetate |
|  | 20.02 | 239.23688 | C_16_H_30_O | Muscone |
|  | 1.251 | 309.11258 | C_19_H_18_O_4_ | 7-Hydroxy-3-(4-methoxyphenyl)-4-propyl-2H-1-benzopyran-2-one |
| Organoheterocyclic compounds (75) | 0.771 | 318.12952 | C_16_H_19_N_3_O_2_S | N-{[5-(tert-butyl)-2-thienyl]carbonyl}-N'-(6-methyl-2-pyridyl)urea |
|  | 0.775 | 326.12669 | C_16_H_18_FN_3_O_2_ | (3R,5S)-5-(3-Cyclopropyl-1,2,4-oxadiazol-5-yl)-1-(4-fluorobenzyl)-3-pyrrolidinol |
|  | 0.792 | 405.11873 | C_20_H_24_O_10_ | (1R,3R,8S,10R,13S,16S,17R)-6,12,17-Trihydroxy-16-methyl-8-(2-methyl-2-propanyl)-2,4,14,19-tetraoxahexacyclo[8.7.2.01,11 03,7 07,11 013,17]nonadecane-5,15,18-trione |
|  | 0.801 | 385.07706 | C_18_H_13_FN_4_O_3_S | N1-(3-fluorophenyl)-2-{[5-(4-nitrophenyl)-2-furyl]methylidene}hydrazine-1-carbothioamide |
|  | 0.806 | 380.97995 | C_17_H_11_Cl_3_N_2_S | N-(2-naphthyl)-N'-(2,4,5-trichlorophenyl)thiourea |
|  | 0.808 | 371.02527 | C_15_H_12_ClFN_2_O_4_S | O1-(4-Chlorobenzoyl)-2-[(4-fluorophenyl)sulfonyl]ethanehydroximamide |
|  | 0.809 | 213.07477 | C_10_H_12_O_5_ | Danielone |
|  | 0.815 | 333.06927 | C_19_H_12_N_2_O_2_S | 4-[2-cyano-2-(2-pyridyl)vinyl]phenyl thiophene-2-carboxylate |
|  | 0.83 | 219.02644 | C_7_H_10_N_2_O_2_S_2_ | Ethyl 4-amino-2-(methylsulfanyl)-1,3-thiazole-5-carboxylate |
|  | 0.831 | 323.07371 | C_14_H_15_ClN_4_OS | N'2-{2-[(4-Chlorophenyl)thio]ethanimidoyl}-1-methyl-1H-pyrrole-2-carbohydrazide |
|  | 0.847 | 218.03286 | C_6_H_10_N_3_O_4_P | 4-Amino-2-methyl-5-(phosphooxymethyl)pyrimidine |
|  | 0.865 | 236.11271 | C_11_H_13_N_3_O_3_ | 4-(dimethylamino)-3-(3-nitropyridin-2-yl)but-3-en-2-one |
|  | 0.871 | 285.08224 | C_14_H_14_N_4_OS | 3-(2,1,3-Benzothiadiazol-4-ylamino)-2-(2,2-dimethylpropanoyl)acrylonitrile |
|  | 0.876 | 203.10265 | C_8_H_14_N_2_O_4_ | Proclavaminic acid |
|  | 0.884 | 349.1352 | C_21_H_20_N_2_OS | N-benzhydryl-N'-(2-methoxyphenyl)thiourea |
|  | 0.889 | 429.12459 | C_20_H_23_N_5_O_2_S_2_ | O1-(2-thienylcarbonyl)-2-({5-[4-(tert-butyl)phenyl]-4-methyl-4H-1,2,4-triazol-3-yl}thio)ethanehydroximamide |
|  | 0.912 | 490.17648 | C_27_H_24_FN_3_O_5_ | 8-(2,4-Dimethoxyphenyl)-2-(3-fluorobenzoyl)-1,3,4,12a-tetrahydropyrazino[2,1-c][1,4]benzodiazepine-6,12(2H,11H)-dione |
|  | 1.121 | 304.99158 | C_15_H_8_F_2_OS_2_ | (2,4-difluorophenyl)[5-(2-thienyl)-2-thienyl]methanone |
|  | 1.149 | 141.01827 | C_6_H_4_O_4_ | Cis-4-Carboxymethylenebut-2-en-4-olide |
|  | 1.195 | 224.09156 | C_11_H_13_NO_4_ | Bendiocarb |
|  | 1.199 | 294.10023 | C_15_H_11_N_5_O_2_ | 6-amino-4-spiro(1,3-dihydro-2-oxo-2H-indol-3-yl)-1,4-dihydropyrano[2,3-c]pyrazole-5-carbonitrile |
|  | 1.218 | 213.12318 | C_10_H_16_N_2_O_3_ | Butabarbital |
|  | 1.225 | 276.14402 | C_16_H_21_NOS | 4-(3,4-dimethylphenyl)-1-thia-4-azaspiro[4.5]decan-3-one |
|  | 1.236 | 312.09491 | C_12_H_19_N_5_OS_2_ | 2-[(5-Amino-1,3,4-thiadiazol-2-yl)thio]-N-(8-methyl-8-azabicyclo[3.2.1]oct-3-yl)acetamide |
|  | 1.246 | 342.11941 | C_19_H_21_NO_3_S | 6-{[(1-benzothiophen-3-ylmethyl)amino]carbonyl}-3,4-dimethylcyclohex-3-ene-1-carboxylic acid |
|  | 1.248 | 522.18256 | C_25_H_30_FN_3_O_6_ | N-{[(2R,3S,4R,5S)-3,4-Dihydroxy-5-{2-[4-(2-methoxyphenyl)-1-piperazinyl]-2-oxoethyl}tetrahydro-2-furanyl]methyl}-3-fluorobenzamide |
|  | 1.251 | 297.11902 | C_16_H_18_N_4_S | N-[1-methyl-1-(2-phenyldiaz-1-enyl)ethyl]-N'-phenylthiourea |
|  | 1.269 | 303.14465 | C_15_H_18_N_4_O_3_ | 2-(benzyl{2-[(1-methyl-1H-pyrazol-5-yl)amino]-2-oxoethyl}amino)acetic acid |
|  | 1.298 | 160.07572 | C_10_H_9_NO | 1H-indene-3-carboxamide |
|  | 1.407 | 334.16068 | C_22_H_23_NS | Tropatepine |
|  | 1.508 | 328.13882 | C_16_H_14_N_4_O_3_ | 7-amino-1,3-dimethyl-2,4-dioxo-5-phenyl-1,3,4,5-tetrahydro-2H-pyrano[2,3-d]pyrimidine-6-carbonitrile |
|  | 1.702 | 311.12363 | C_14_H_18_N_2_O_6_ | Ethyl 2-{[(ethoxycarbonyl)amino]carbonyl}-3-[(2-furylmethyl)amino]acrylate |
|  | 1.715 | 154.99879 | C_6_H_4_O_5_ | 2,5-Furandicarboxylic acid |
|  | 2.362 | 237.1234 | C_12_H_16_N_2_O_3_ | 3-Morpholino-4-tetrahydro-1H-pyrrol-1-ylcyclobut-3-ene-1,2-dione |
|  | 3.107 | 367.14982 | C_17_H_22_N_2_O_7_ | 2-{2-[5-(Ethoxycarbonyl)-2-morpholinoanilino]-2-oxoethoxy}acetic acid |
|  | 3.317 | 132.08081 | C_9_H_9_N | Skatole |
|  | 3.331 | 144.08084 | C_10_H_9_N | 6-Methylquinoline |
|  | 3.588 | 146.06007 | C_9_H_7_NO | 4-Indolecarbaldehyde |
|  | 3.701 | 203.0826 | C_11_H_12_N_2_O_2_ | DL-Tryptophan |
|  | 3.789 | 342.08776 | C_20_H_13_N_3_O_3_ | Violacein |
|  | 4.541 | 423.18746 | C_23_H_26_N_4_O_2_S | N-({(2R,4S,5R)-5-[6-(2-Furyl)-2-methyl-4-pyrimidinyl]-1-azabicyclo[2.2.2]oct-2-yl}methyl)-2-(2-thienyl)acetamide |
|  | 4.933 | 430.22835 | C_22_H_31_N_5_O_2_S | 2-(1-Adamantyl)-6-{[(5-hydroxy-4-pentyl-4H-1,2,4-triazol-3-yl)thio]methyl}pyrimidin-4-ol |
|  | 5.022 | 457.1925 | C_22_H_34_N_2_O_4_S | 2-[(1S,4S,5S)-5-Isopropyl-2-methyl-4-{[(methylsulfonyl)amino]methyl}-2-cyclohexen-1-yl]-N-(4-methoxybenzyl)acetamide |
|  | 5.643 | 304.16551 | C_16_H_23_N_3_O_4_ | 1-{4-[(2R,3R)-3-(Hydroxymethyl)-4-methyl-5-oxo-2-morpholinyl]phenyl}-3-isopropylurea |
|  | 5.852 | 370.08895 | C_17_H_14_F_3_NO_5_ | 4-oxo-4-{4-[4-(trifluoromethoxy)phenoxy]anilino}butanoic acid |
|  | 5.902 | 302.14987 | C_16_H_19_N_3_O_3_ | O1-[(5-methylisoxazol-3-yl)carbonyl]-4-(tert-butyl)benzene-1-carbohydroximamide |
|  | 5.993 | 427.11055 | C_23_H_17_F_3_N_2_OS | 2-{4-[4-(2,4-Dimethylphenyl)-1,3-thiazol-2-yl]phenoxy}-5-(trifluoromethyl)pyridine |
|  | 6.123 | 282.09709 | C_13_H_15_NO_6_ | Ethyl 3-hydroxy-4,6-dimethoxy-2-oxoindoline-3-carboxylate |
|  | 6.865 | 449.17836 | C_22_H_23_F_3_N_4_O_3_ | (3R,5S)-1-[4-(Dimethylamino)benzyl]-5-{3-[4-(trifluoromethoxy)phenyl]-1,2,4-oxadiazol-5-yl}-3-pyrrolidinol |
|  | 6.867 | 268.13313 | C_12_H_15_ClN_4_ | 6-Chloro-N2,N3-diethylquinoxaline-2,3-diamine |
|  | 6.981 | 377.16067 | C_21_H_22_N_4_O_3_ | (3S)-N-(2-Methoxyphenyl)-3-[5-(4-methylphenyl)-1,3,4-oxadiazol-2-yl]-1-pyrrolidinecarboxamide |
|  | 7.176 | 336.19161 | C_17_H_25_N_3_O_4_ | (3R,4R)-N-Ethyl-4-hydroxy-3-[(4-methoxybenzoyl)amino]-1-azepanecarboxamide |
|  | 7.367 | 262.07436 | C_13_H_12_ClN_3_O | 2-[2-(4-chlorophenyl)-2-methylhydrazono]-3-cyclopropyl-3-oxopropanenitrile |
|  | 7.61 | 273.07566 | C_15_H_12_O_5_ | Alternariolmethylether |
|  | 7.655 | 429.17662 | C_21_H_26_N_4_O_6_ | N-{[(2R,3S,4R,5S)-3,4-Dihydroxy-5-{2-oxo-2-[4-(2-pyridinyl)-1-piperazinyl]ethyl}tetrahydro-2-furanyl]methyl}-2-furamide |
|  | 8.243 | 284.12802 | C_17_H_17_NO_3_ | (2E)-3-(4-Hydroxyphenyl)-N-[2-(4-hydroxyphenyl)ethyl]acrylamide |
|  | 8.504 | 314.1384 | C_19_H_15_N_5_ | 4-(2,3-dihydro-1H-indol-1-yl)-1-phenyl-1H-pyrazolo[3,4-d]pyrimidine |
|  | 10.273 | 268.13312 | C_17_H_17_NO_2_ | 6,7-Dimethoxy-1-phenyl-3,4-dihydroisoquinoline |
|  | 10.618 | 195.13806 | C_12_H_18_O_2_ | Sedanolide |
|  | 11.902 | 280.0967 | C_17_H_13_NO_3_ | Alpha-Phthalimidopropiophenone |
|  | 12.145 | 343.2489 | C_19_H_36_O_5_ | Siegesbeckin A |
|  | 13.579 | 308.12802 | C_19_H_17_NO_3_ | N-(2,3-Dihydro-1-benzofuran-5-ylmethyl)-2H-chromene-3-carboxamide |
|  | 14.188 | 595.28911 | C_29_H_38_N_6_O_5_ | 2-{(2S,3R,4S,5R)-5-[(4-{[Benzyl(methyl)amino]methyl}-1H-1,2,3-triazol-1-yl)methyl]-3,4-dihydroxytetrahydro-2-furanyl}-1-[4-(2-methoxyphenyl)-1-piperazinyl]ethanone |
|  | 14.484 | 476.27724 | C_30_H_37_NO_4_ | Longichalasin B |
|  | 15.031 | 433.23495 | C_24_H_28_N_6_O_2_ | N-{[(1S,4S,6S)-6-Isopropyl-3-methyl-4-{[5-(4-pyridinyl)-1,3,4-oxadiazol-2-yl]methyl}-2-cyclohexen-1-yl]methyl}-2-pyrazinecarboxamide |
|  | 15.103 | 431.22034 | C_24_H_28_N_6_O_2_ | (3R,4S,6R)-N-(2-Methoxybenzyl)-6-{[4-(2-pyridinyl)-1H-1,2,3-triazol-1-yl]methyl}quinuclidine-3-carboxamide |
|  | 15.137 | 507.27264 | C_26_H_40_N_4_O_4_ | N-[(1S,2S,8S,8aS)-8-Hydroxy-7-{(2S)-1-[(2R)-2-(methoxymethyl)-1-pyrrolidinyl]-1-oxo-2-propanyl}-1,4a-dimethyldecahydro-2-naphthalenyl]-5-pyrimidinecarboxamide |
|  | 15.34 | 440.27716 | C_25_H_39_NO_4_ | (7*E*)-12-Hydroxy-3-isobutyl-13-methoxy-4,5,8-trimethyl-3,3a,4,6a,9,10,11,12,13,14-decahydro-1H-cycloundeca[d]isoindole-1,15(2H)-dione |
|  | 15.772 | 425.28957 | C_25_H_36_N_4_O_2_ | 2-{(3R,4S)-3-[(5-Butyl-1,2-oxazol-3-yl)methyl]-4-piperidinyl}-1-(4-phenyl-1-piperazinyl)ethanone |
|  | 15.773 | 627.33487 | C_31_H_43_N_3_O_8_ | Tanespimycin |
|  | 17.088 | 433.23257 | C_22_H_30_N_6_O_2_ | N-Benzyl-3-[(2S,5aS,8aR)-6-(1H-imidazol-2-ylmethyl)-1-methyl-5-oxodecahydropyrrolo[3,2-E][1,4]diazepin-2-yl]propanamide |
|  | 17.155 | 647.39681 | C_40_H_56_O_7_ | Carissin |
|  | 17.41 | 419.25555 | C_24_H_32_N_6_O_2_ | N-{[(1S,4S,6S)-6-Isopropyl-3-methyl-4-{[5-(1-methyl-1H-imidazol-5-yl)-1,3,4-oxadiazol-2-yl]methyl}-2-cyclohexen-1-yl]methyl}-6-methoxy-3-pyridinamine |
|  | 0.921 | 470.15103 | C_24_H_25_N_3_O_5_ | 4-Cyano-N-{4-[(2R,3R)-3-(hydroxymethyl)-5-oxo-4-(tetrahydro-2H-pyran-4-yl)-2-morpholinyl]phenyl}benzamide |
|  | 4.501 | 390.15468 | C_20_H_20_O_7_ | 4-[3-(4-hydroxy-3-methoxybenzoyl)-2,3-dimethyloxirane-2-carbonyl]-2-methoxyphenol |
| others (19) | 0.861 | 248.11278 | C_10_H_17_NO_6_ | Linamarin |
|  | 1.284 | 347.09835 | C_15_H_16_N_4_O_6_ | Musca-aurin-VII |
|  | 1.173 | 175.02489 | C_6_H_8_O_6_ | Ascorbic acid |
|  | 1.196 | 308.09086 | C_10_H_17_N_3_O_6_S | L-Glutathione |
|  | 16.086 | 501.22315 | C_27_H_32_F_6_O_2_ | 26,26,26,27,27,27-hexafluoro-25-hydroxy-16,17,23,23,24,24-hexadehydrovitamin D3 |
|  | 0.806 | 223.02447 | Rn | Radon-222 |
|  | 0.783 | 212.8423 | K_2_O_4_S | Potassium sulfate |
|  | 0.798 | 212.85175 | HK_2_O_4_P | Potassium dibasic phosphate |
|  | 0.791 | 565.0488 | C_15_H_24_N_2_O_17_P_2_ | Uridine 5'-diphosphogalactose |
|  | 0.901 | 264.10762 | C_10_H_17_NO_7_ | 4-O-(beta-L-Arabinofuranosyl)-(2S,4S)-4-hydroxyproline |
|  | 3.632 | 298.09669 | C_11_H_15_N_5_O_3_S | 5'-S-Methyl-5'-thioadenosine |
|  | 7.5 | 229.08594 | C_14_H_12_O_3_ | Resveratrol |
|  | 14.9 | 300.28954 | C_18_H_37_NO_2_ | D-Sphingosine |
|  | 15.022 | 318.30004 | C_18_H_39_NO_3_ | 2-Amino-1,3,4-octadecanetriol |
|  | 15.516 | 407.27907 | C_24_H_38_O_5_ | 3alpha,7alpha-Dihydroxy-12-oxo-5beta-cholanate |
|  | 8.684 | 263.12855 | C_15_H_20_O_4_ | (+/-)-Abscisic acid |
|  | 7.821 | 491.11917 | C_23_H_24_O_12_ | Aurantio-obtusin beta-D-glucoside |
|  | 0.806 | 232.89263 | H_4_CaO_8_P_2_ | Monobasic calcium phosphate |
|  | 1.199 | 213.12302 | C_8_H_8_O_5_._2_Na | Endothal-disodium |
| Phenylpropanoids (13) | 1.206 | 450.1969 | C_19_H_28_O_11_ | 2-(4-Hydroxyphenyl)ethyl 6-O-[(2R,3R,4R)-3,4-dihydroxy-4-(hydroxymethyl)tetrahydro-2-furanyl]-beta-D-glucopyranoside |
|  | 6.68 | 191.03501 | C_10_H_8_O_4_ | Isoscopoletin |
|  | 4.2 | 339.07208 | C_15_H_16_O_9_ | Esculin |
|  | 5.03 | 177.0194 | C_9_H_6_O_4_ | Esculetin |
|  | 6.203 | 147.04407 | C_9_H_8_O_3_ | 4-Coumaric acid |
|  | 6.203 | 147.04408 | C_9_H_6_O_2_ | Coumarin |
|  | 6.593 | 193.04956 | C_10_H_8_O_4_ | Scopoletin |
|  | 6.085 | 163.07533 | C_10_H_10_O_2_ | Methyl cinnamate |
|  | 6.29 | 163.04017 | C_9_H_8_O_3_ | 3-Coumaric acid |
|  | 7.267 | 519.18671 | C_26_H_32_O_11_ | Pinoresinol 4-O-glucoside |
|  | 19.312 | 161.0598 | C_10_H_10_O_3_ | 4-Methoxycinnamic acid |
|  | 5.934 | 368.07452 | C_16_H_16_O_10_ | 3-hydroxy-2-{[(2E)-3-(4-hydroxyphenyl)prop-2-enoyl]oxy}-3-(methoxycarbonyl)pentanedioic acid |
|  | 1.196 | 295.10339 | C_11_H_18_O_9_ | Tuliposide B |
| Polyketides (4) | 15.076 | 701.37126 | C_35_H_56_O_14_ | Chalcomycin |
|  | 3.435 | 593.13047 | C_30_H_26_O_13_ | Tribuloside |
|  | 0.855 | 487.21383 | C_30_H_32_O_6_ | Flemiphilippinin A |
|  | 14.126 | 579.29285 | C_27_H_47_O_11_P | PKODiA-PA |
| Terpenoids (13) | 1.225 | 258.13337 | C_12_H_16_O_5_ | 5-hydroxy-4-[3-(2-hydroxypropan-2-yl)oxiran-2-yl]-1-methyl-7-oxabicyclo[4.1.0]hept-3-en-2-one |
|  | 1.45 | 279.1009 | C_18_H_14_O_3_ | Dihydrotanshinone I |
|  | 0.77 | 273.14426 | C_15_H_22_O_3_ | 6-hydroxy-4a-(hydroxymethyl)-5-methyl-3-(prop-1-en-2-yl)-2,3,4,4a,5,6,7,8-octahydronaphthalen-2-one |
|  | 7.486 | 216.12307 | C_10_H_14_O_4_ | (-)-Camphanic acid |
|  | 8.605 | 310.20115 | C_17_H_24_O_4_ | (2E)-3-[(1R,4S,7R,7aR)-1-(acetyloxy)-3,7-dimethyl-2,4,5,6,7,7a-hexahydro-1H-inden-4-yl]-2-methylprop-2-enoic acid |
|  | 10.619 | 149.13258 | C_11_H_16_ | Ectocarpen |
|  | 14.122 | 579.29274 | C_34_H_44_O_9_ | Salannin |
|  | 14.777 | 555.293 | C_32_H_42_O_8_ | Acrovestone |
|  | 15.378 | 537.30343 | C_29_H_44_O_9_ | Rhodexin A |
|  | 16.02 | 869.49432 | C_45_H_72_O_16_ | Dioscin |
|  | 17.628 | 353.26824 | C_21_H_36_O_4_ | Montanol |
|  | 17.814 | 366.30023 | C_22_H_36_O_3_ | 3-Methyl-5-(5,5,8a-trimethyl-2-methylene-7-oxodecahydro-1-naphthalenyl)pentyl acetate |
|  | 6.903 | 401.15711 | C_20_H_26_O_7_ | 5-(furan-3-yl)-1',9'-dihydroxy-12'-(hydroxymethyl)-6'-methyl-3'-oxaspiro[oxolane-3,7'-tricyclo[6.3.1.0]dodecane]-2-one |
